# Light‐Activatable Nitric Oxide Release via Intramolecular Electron Transfer for Tumor Pyroptosis Induction

**DOI:** 10.1002/advs.202522486

**Published:** 2026-05-25

**Authors:** Chuangxin Zhang, Ruipeng Li, Yunxia Wang, Feng Liu, Liheng Feng

**Affiliations:** ^1^ School of Chemistry and Chemical Engineering Shanxi University Taiyuan China; ^2^ Future Organic Optoelectronics Research Center Global Institute of Future Technology Shanghai Jiao Tong University Shanghai China

**Keywords:** intramolecular electron transfer, nitric oxide release, peroxynitrite generation, photocatalysis, pyroptosis

## Abstract

Developing long‐wavelength‐activated nitric oxide (NO) donors has attracted increasing attention for achieving precise photo‐controlled NO release with deep tissue penetration and minimal phototoxicity, yet this remains a major challenge. Herein, we report a deep‐red light‐triggered NO‐releasing molecule (DBTBT‐NO) via a novel photoexcitation‐mediated electron transfer process to induce abundant peroxynitrite generation. The DBTBT‐NO can be activated by deep‐red light to release NO, which is facilitated by photoinduced intramolecular charge separation. Meanwhile, superoxide anions generated during photolysis rapidly react with NO to produce massive peroxynitrite in mitochondria, which induces tumor cell pyroptosis at a low light dose of 15 J/cm^2^ and a low concentration of 0.4 µm. The robust pyroptosis triggered by DBTBT‐NO effectively inhibits tumor growth and metastasis in mice with excellent biosafety. This is the first report on boosting peroxynitrite generation through long‐wavelength photolysis of benzisothiadiazole‐based *N*‐nitrosanilines via a distinct mechanism. This work provides a new approach to fabricate unimolecular peroxynitrite photoinitiators with high pyroptosis induction efficiency, facilitating simplified and improved photoimmunotherapy.

## Graphical abstract

This work proposes a novel strategy to fabricate unimolecular pyroptosis photoinitiators activated by deep‐red light at low light doses. It relies on a photosensitization mechanism to trigger nitric oxide (NO) release from benzisothiadiazole‐based *N*‐nitrosanilines without modification of nitro moieties, thereby boosting pyroptosis‐mediated photoimmunotherapy and showing considerable promise for biomedical applications.

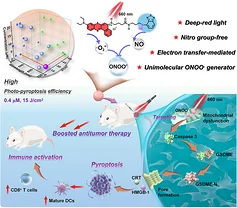

## 1 Introduction

Nitric oxide (NO) is a critical signaling molecule that plays central roles in various physiological processes, including inflammation, wound healing, and tumor progression [1, 2, 3, 4, 5]. Additionally, NO can react with superoxide anion (O_2_ **^·^** ^−^) to generate peroxynitrite (ONOO^−^), a multitarget cytotoxic agent [6, 7]. However, the therapeutic applications of NO are limited by nonspecific biodistribution, unexpected release, and off‐target effects, due to the short half‐life [8, 9]. Facing such challenges, photo‐controlled NO release undoubtedly provides a promising strategy due to the non‐invasive and spatiotemporal control feature of light. Numerous studies have demonstrated the advantages of light‐controlled behavior in antitumor therapy [10, 11, 12]. Hence, photo‐activatable NO‐releasing molecules serving as NO donors are continually synthesized and investigated [13, 14]. Nevertheless, early developed photo‐activatable NO donors mostly response to ultraviolet (UV) or near‐UV visible light, which greatly hampers the biomedical application due to the poor tissue penetration and high phototoxicity. To gain deep tissue penetration and minimal photodamage, researchers try to concentrate on developing long‐wavelength‐responsive NO donors, especially in the red and near‐infrared (NIR) windows (600–900 nm) [15, 16, 17, 18, 19, 20, 21]. Among these efforts, Hu and co‐workers reported a photoredox catalysis strategy to take advantage of the electron transfer between activated photocatalysts (PCs) and NO donors for activation of NO donors that cannot be activated by long‐wavelength light [22, 23, 24, 25]. This electron transfer‐based catalytic mechanism can reduce the required excitation energy while ensuring the loading rate of NO donors, which inspires researchers to develop more NO donors excited at low energy.

As the important downstream product of NO, ONOO^−^ plays important roles in therapeutic application, particularly in antitumor treatment. It has been well documented that ONOO^−^ can induce ferroptosis or pyroptosis to trigger immunogenic cell death (ICD) in tumor cells, thereby effectively enhancing antitumor immunotherapy by remodeling the immunosuppressive tumor microenvironment [26, 27, 28, 29, 30]. Given that ONOO^−^ is short‐lived and transient, and is generated via the diffusion‐controlled reaction between NO and O_2_ **^·^** ^−^, its therapeutic utility strongly relies on effective NO release and spatiotemporally synchronous formation with O_2_ **^·^** ^−^. Despite the encapsulation of various NO donors and O_2_ **^·^** ^−^ generators into nanoplatforms, the production rate of ONOO^−^ is still slow, owing to the mismatched release kinetics and large reaction distance between NO and O_2_ **^·^** ^−^. This results in the requirement of prolonged irradiation duration and high light intensity. Therefore, developing a long‐wavelength‐responsive NO donors integrated with synchronous O_2_ **^·^** ^−^ generation ability is expected to accelerate ONOO^−^ production and maximize the therapeutic functions of NO under mild irradiation conditions, whether for improved antitumor photoimmunotherapy or other therapeutic applications. Such a system is urgently pursued by researchers, yet remains greatly challenging to realize.

Herein, we report a novel strategy based on a photosensitization mechanism to trigger NO release and boost ONOO^−^ generation under deep‐red light irradiation, achieving highly efficient tumor cell pyroptosis at a low light dose (15 J/cm^2^) and low concentration (0.4 µm). By covalently conjugating Nile blue derivatives with benzisothiadiazole‐based *N*‐nitrosaniline via flexible spacers, the synthesized molecules (DBTBT‐NO) enable the NO part to release from the molecular skeleton via a photoinduced electron transfer process. Since the photolysis process simultaneously generates O_2_ **^·^** ^−^, the rapid reaction between O_2_ **^·^** ^−^ and NO induces abundant ONOO^−^ production. Thus, this system not only serves as a novel deep‐red light‐activatable NO donor but also an ONOO^−^ donor. DBTBT‐NO can self‐assemble into nanoparticles in aqueous solution and efficiently trigger mitochondria‐targeted pyroptosis as well as robust immune responses. In vivo experiments demonstrate that light‐activated DBTBT‐NO successfully inhibits tumor growth and metastasis in mice through potent tumor cell ablation and antitumor immune activation, with favorable biosafety. This work provides a new paradigm for deep‐red light‐triggered NO release from benzisothiadiazole‐based *N*‐nitrosanilines without nitro moieties, which also acts as a unimolecular ONOO^−^ generator. This strategy thereby inspires researchers to develop more efficient and concise molecular design principles for ONOO^−^ generation.

## 2 Results and Discussions

### 2.1 Rational Design and Synthesis

**SCHEME 1:**
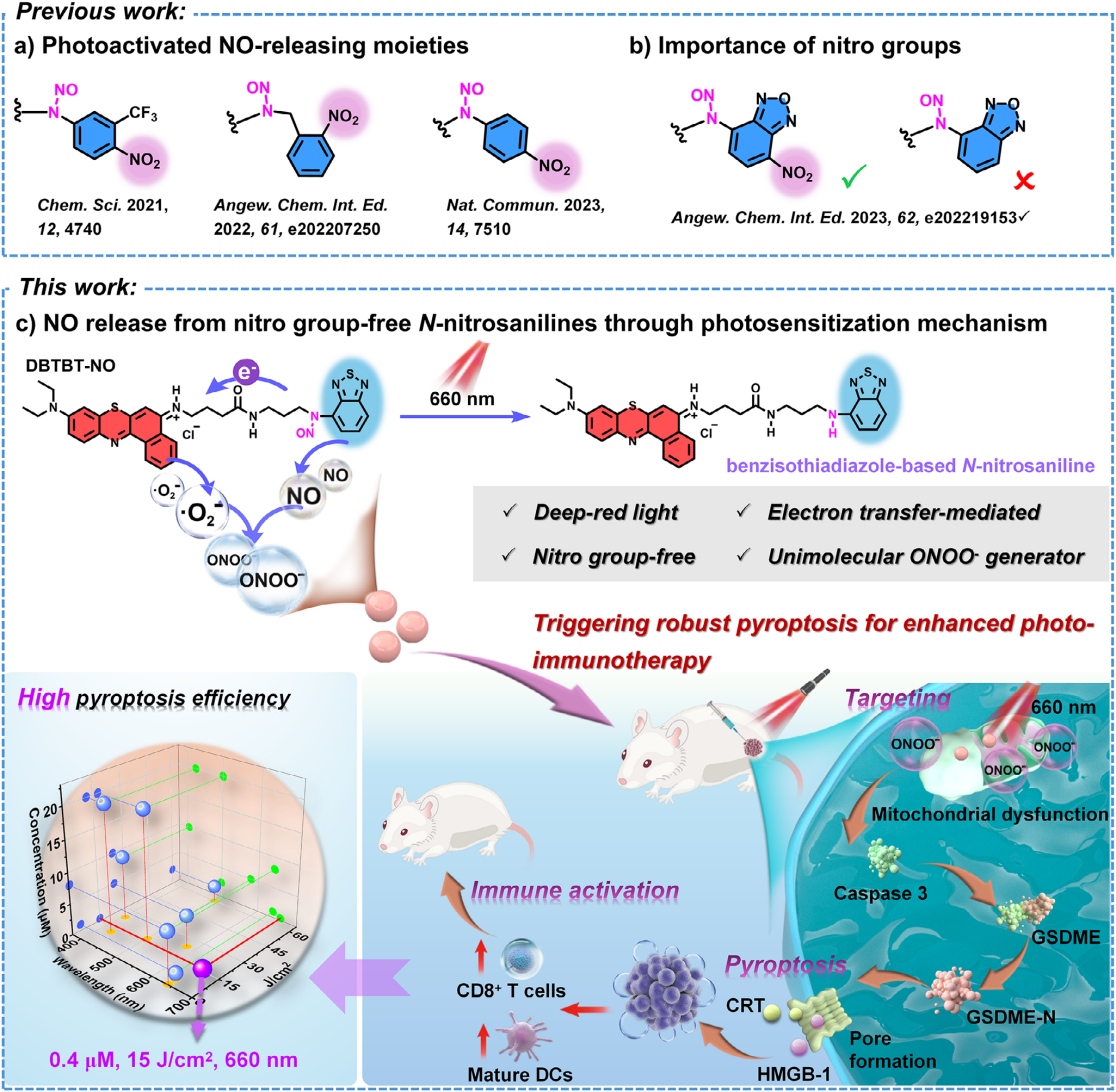
Schematic illustration of (a) the structures of current photoactivated NO‐releasing moieties and (b) an important demonstration of the nitro group in the photocatalysis process. (c) Schematic illustration of proposed deep‐red light‐direct activation of NO release from nitro group‐free *N*‐nitrosanilines through photosensitization mechanism and its application for targeting pyroptosis‐mediated enhanced photo‐immunotherapy.

To achieve deep tissue penetration and minimal phototoxicity, developing long‐wavelength‐activated NO donors becomes imperative. Both Sortino and Hu groups are dedicated to investigating various NO‐releasing moieties and mechanisms (Scheme 1a) [20, 21, 22, 23, 24, 25, 31, 32]. Most applied NO‐releasing moieties are *N*‐nitrosamine that are covalently linked to aromatic rings modified with electron‐withdrawing groups, such as nitro groups. Recently, the Hu group reported a photoredox catalysis strategy to activate nitrobenzofurazan‐based NO donors (NBDNO) to release NO with near‐infrared light by encapsulation with Nile blue derivatives as photocatalysts, which avoids the prior deoxygenation process or addition of sacrificial agents [33]. They also synthesized a counterpart (BNNO) without a nitro group to demonstrate the importance of the nitro group for photocatalysis process (Scheme 1b). Although nitro group‐containing compounds exhibit wide biological applications, compelling evidence indicates that their use can lead to severe toxicity, which is undoubtedly a major reason for their cautious use or avoidance in pharmaceutical development [34]. Herein, it is necessary and challenging to activate nitro group‐free *N*‐nitrosanilines‐based NO donors.

Inspired by the above investigation, we covalently linked a Nile blue derivative with a benzisothiadiazole‐based *N*‐nitrosaniline group directly through a flexible spacer to construct a novel type of NO donor (DBTBT‐NO) without the need of modification of nitro groups (Scheme 1c). Benthiadiazole has a similar structure to benzooxadiazole by substituting the O atom with an S atom, which acts as a key structural moiety in the molecular design for medicinal chemistry [35, 36]. Under deep‐red light irradiation, the NO donor can perform an intramolecular electron transfer process to trigger the NO release. Since the Nile blue derivative can generate O_2_ **^·^** ^−^ in the photolysis process, the rapid reaction of O_2_ **^·^** ^−^ and NO is supposed to fast generate massive ONOO^−^ that is capable of triggering pyroptosis. It is demonstrated that the wavelength (660 nm) employed in this work is longer, whereas the light intensity (15 J/cm^2^) and reagent concentration (0.4 µm) are lower than those reported in previous pyroptosis‐triggering studies. Such an unimolecular photoinitiator of pyroptosis mediated by ONOO^−^ is expected to enhance anti‐tumor immunogenicity by promoting mature of dendritic cells (DCs) and CD8^+^ T cell infiltration, finally improving the photo‐immunotherapy. The synthetic route of DBTBT‐NO is shown in Figure S1, and all the relevant molecules are characterized by nuclear magnetic resonance (NMR) and high‐resolution mass spectrometry (HR‐MS; Figures S2–S9).

### 2.2 Property Characterization of DBTBT‐NO

**FIGURE 1:**
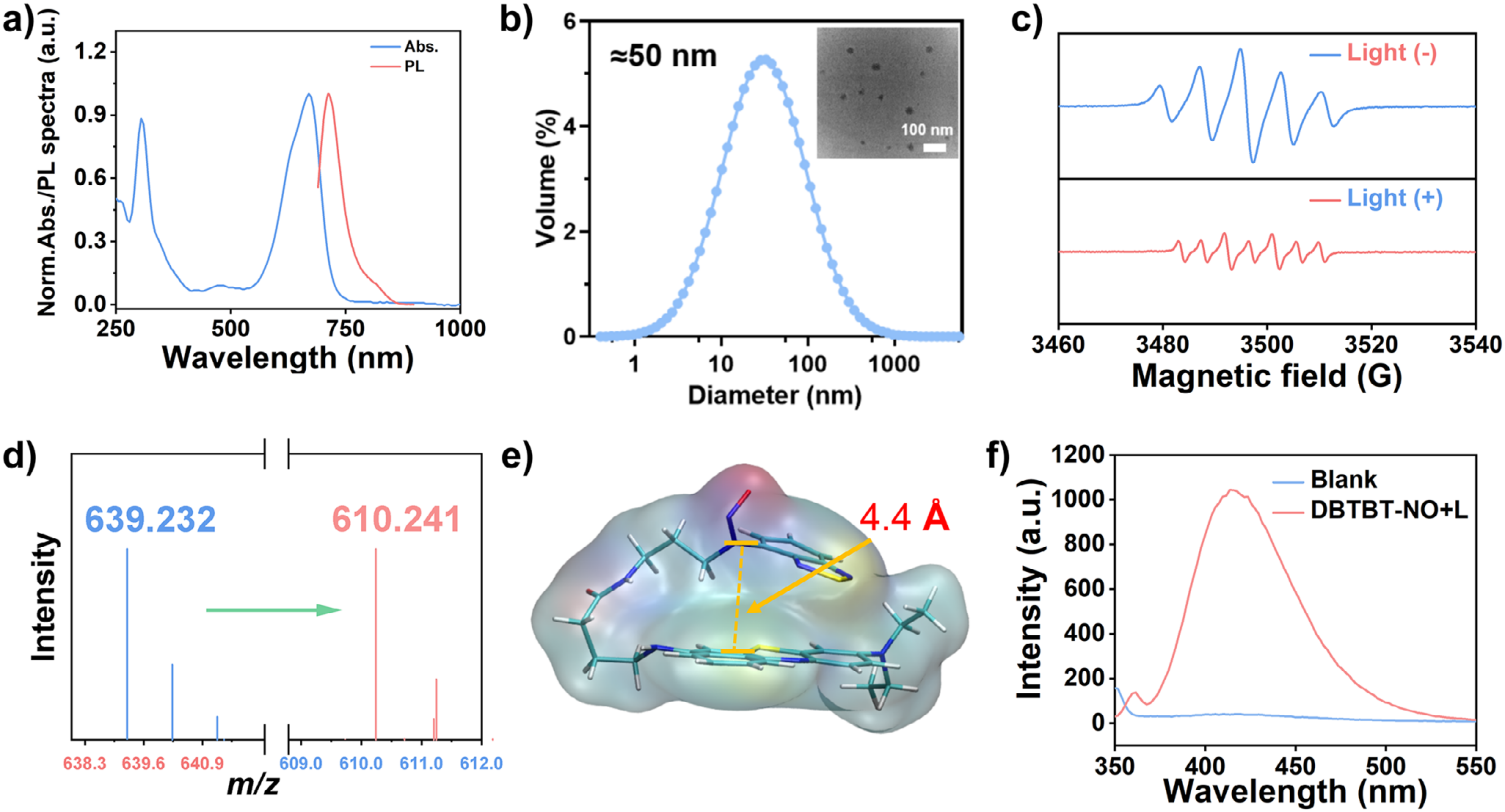
(a) Normalized UV–vis absorption and fluorescence spectra of DBTBT‐NO in aqueous solution. (b) Hydrodynamic diameters and TEM image of DBTBT‐NO nanoparticles. (c) EPR spectra of DBTBT‐NO (5 µm) with or without 660 nm irradiation in the presence of PTIO (40 µm). (d) HR‐MS of DBTBT‐NO before and after 660 nm irradiation. (e) Calculated distance between Nile blue derivative moiety and *N*‐nitrosaniline group. (f) The fluorescence changes at 460 nm of DBTBT‐NO aqueous solution in the presence of L‐tyrosine (0.5 mM) with or without 660 nm irradiation (1 W/cm^2^, 5 min).

The photophysical properties of the synthesized DBTBT‐NO were first investigated. As shown in Figure 1a, DBTBT‐NO exhibited strong absorption within the phototherapeutic window, with an intense peak at 670 nm. Under excitation of the maximum wavelength, DBTBT‐NO showed an intense fluorescence emission peak at 714 nm, extending to the near‐infrared region. Owing to hydrophobic and electrostatic interactions, DBTBT‐NO self‐assembled into nanoparticles in aqueous solution with diameters of ∼50 nm (Figure 1b) and positive charges (Figure S10), as evidenced by dynamic light scattering (DLS). Transmission electron microscopy (TEM) images showed that the nanoparticles had a uniform spherical morphology. Under irradiation of 660 nm laser, the NO release was detected in DBTBT‐NO aqueous solution by using electron paramagnetic resonance (EPR) spectra with 2‐phenyl‐4,4,5,5‐tetramethyl‐imidazoline‐1‐oxyl 3‐oxide (PTIO) as a spin‐trapping agent (Figure 1c). HR‐MS spectra indicated that the molecular weight of photolysis product was 610.241 (Figure 1d), which agreed well with that of non‐nitrosated compound DBTBT‐NH. This further demonstrated that red light irradiation triggered the NO release from DBTBT‐NO. Meanwhile, O_2_ **^·^** ^−^ was also detected by using dihydroethidium (DHE) as a fluorescent probe (Figure S11). Considering that the Nile blue derivative moiety is very close to the *N*‐nitrosaniline group (4.4 Å, Figure 1e), the simultaneously generated NO and O_2_ **^·^** ^−^ can fast react to generate ONOO^−^. As expected, ONOO^−^ was identified to intensively generate in the DBTBT‐NO aqueous solution by detecting fluorescence intensity changes of L‐tyrosine dimerization (Dityr) at 406 nm (Figure 1f), and the quantitative analysis result revealed that 55 µm of ONOO^−^ was generated within 5 min of red light irradiation (Figure S12).

### 2.3 NO Release Mechanism of DBTBT‐NO

**FIGURE 2:**
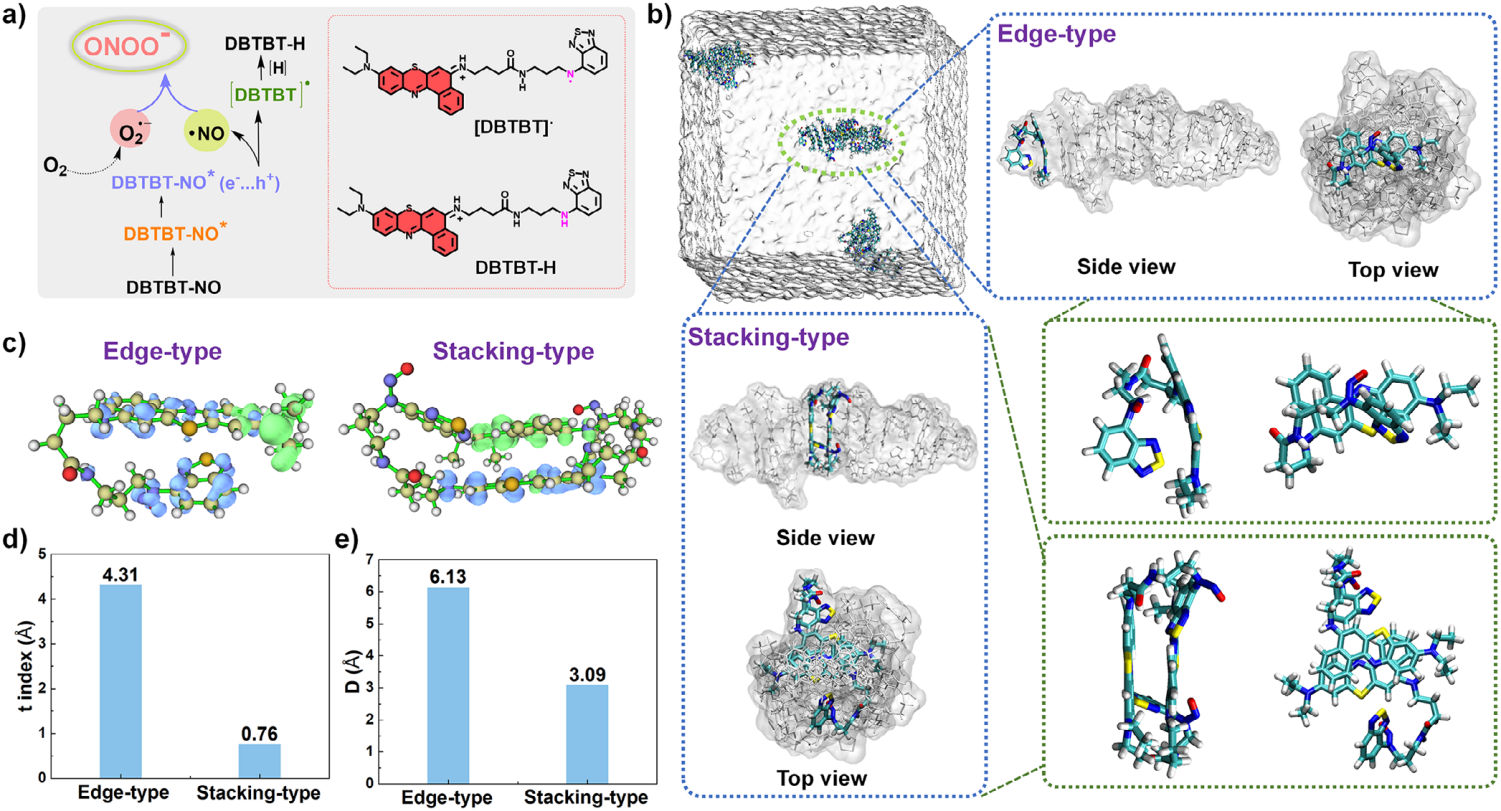
(a) Proposed photosensitization mechanism of NO release from benzisothiadiazole‐based *N*‐nitrosaniline group and concurrent generation of ONOO^−^. (b) Representative molecular packing structures of DBTBT‐NO clusters derived from 600 ns molecular MD simulations, revealing edge‐type and stacking‐type stacking configurations. (c) Spatial distribution of photogenerated electrons (e^−^, green) and holes (h^+^, blue) on DBTBT‐NO edge‐type and stacking‐type. (d), (e) Quantitative analysis of charge‐transfer character using the charge‐transfer index (t) and charge‐transfer distance (D) between photogenerated electron‐hole pairs for each configuration.

Considering the Nile blue derivative could serve as PC to activate NO release of nitrobenzofurazan‐based NO donors [33], we proposed that the Nile blue moiety could promote the NO release of benzisothiadiazole‐based *N*‐nitrosaniline in DBTBT‐NO under light irradiation. The proposed mechanism of NO release was illustrated in Figure 2a. Upon deep‐red light irradiation, DBTBT‐NO was excited to its singlet excited state (DBTBT‐NO^*^). The excited state facilitated photoinduced intramolecular charge separation, generating an electron ‐hole (e^−^/h^+^) pair, which weakened the N─N bond and triggered homolytic cleavage to release NO and the radical intermediate [DBTBT]**^·^**. The [DBTBT]**^·^** subsequently abstracted [H] from the surrounding medium to yield DBTBT‐H. Simultaneously, the photogenerated electron reduced O_2_ to O_2_ **^·^** ^−^ via a type I photochemical pathway. Hence, the co‐generated NO and O_2_ **^·^** ^−^ reacted rapidly in situ to produce the highly reactive species ONOO^−^.

To further confirm the proposed mechanism, molecular dynamics (MD) simulations were performed to uncover the aggregation behaviors of DBTBT‐NO in solution. DBTBT‐NO spontaneously formed aggregates after 600 ns of MD simulation in solution (Figure 2b), in contrast to its random distribution at the initial state (Figure S13). Two typical molecular arrangements were identified within the aggregates. The first was the edge‐type arrangement observed at the periphery of the cluster, in which individual DBTBT‐NO molecules adopted a U‐shaped conformation that brought the Nile blue moiety and the *N*‐nitrosaniline unit into close spatial proximity, thereby facilitating intramolecular photoinduced electron transfer. The second was the stacking‐type arrangement, in which two molecules associated through close intermolecular π‐π stacking interactions, providing a structural basis for intermolecular electron transfer. To determine which electron transfer pathway more readily drove NO release, the hole‐electron distributions of two molecular arrangements were calculated (Figure 2c). The charge‐transfer index (t index), which quantifies the spatial separation between the hole and electron distributions, was found to be 4.31 for the edge‐type (D of 6.13), significantly greater than 0.76 for the stacking‐type (D of 3.09), indicating that intramolecular charge separation was more efficient (Figure 2d,e). Consistently, the Gibbs free energy barrier (ΔG) for NO release was calculated to be 24.66 kcal/mol for the edge‐type, lower than that for the stacking‐type (34.13 kcal/mol). Taken together, these results demonstrate that intramolecular electron transfer in the U‐shaped monomer conformation is the dominant pathway, lowering the NO release barrier and facilitating photoinduced NO generation.

### 2.4 Cell Internalization and Intracellular NO Release and ONOO^−^ Generation

**FIGURE 3:**
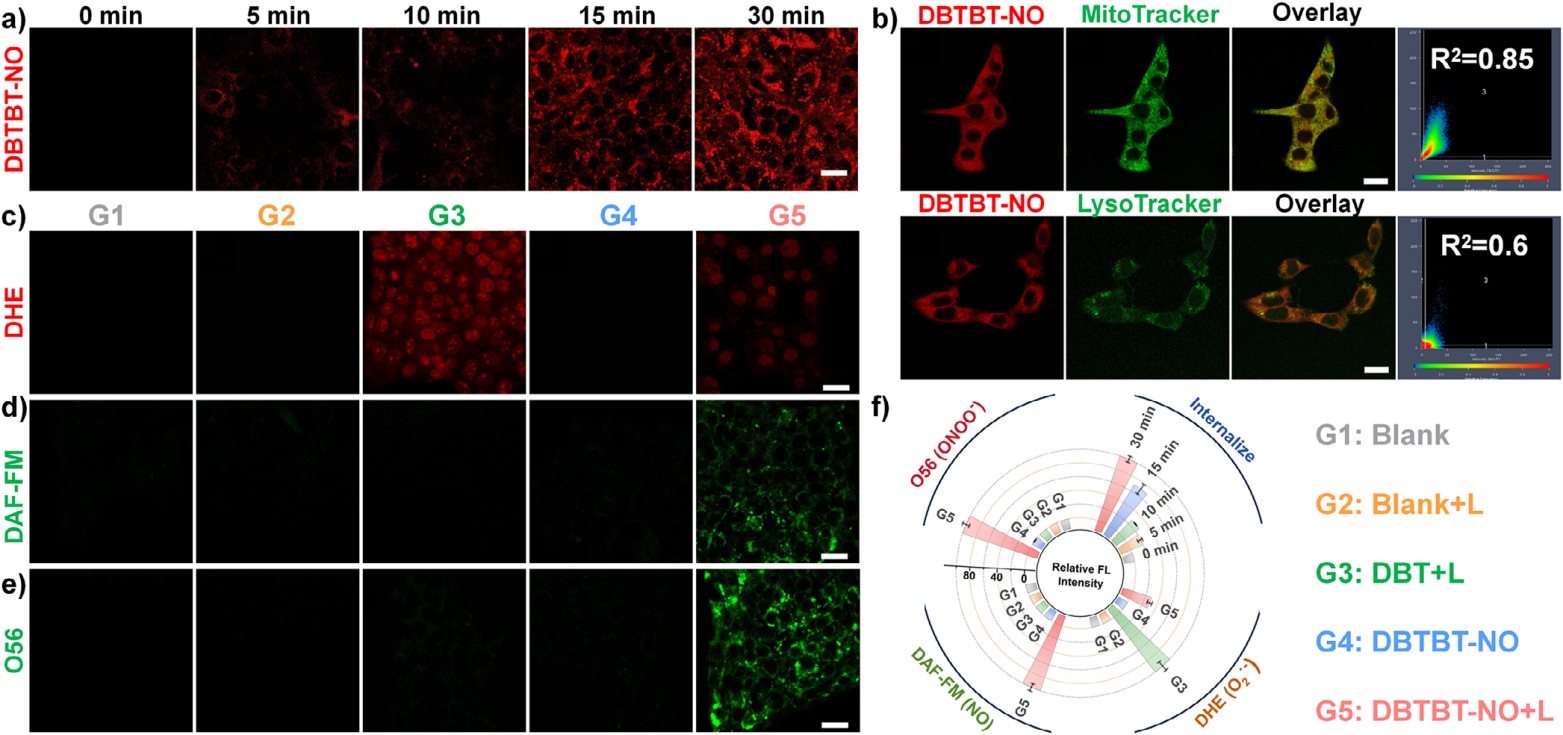
(a) CLSM images of 4T1 cells incubated with DBTBT‐NO for different times. (b) Co‐localization images of DBTBT‐NO and Mito–Tacker (100 nM) or Lyso–Tacker (100 nM) in 4T1 cells. (c–e) The intracellular levels of O_2_ **^·^** ^−^, NO and ONOO^−^ in 4T1 cells after different treatments by using DHE (10.0 µm), DAF‐FM DA (5.0 µm), and O56 (2.0 µm) as probes, respectively. (f) Quantitative analysis of (a–e). The concentration of DBTBT‐NO was 0.4 µm in all the above experiments (660 nm light irradiation: 0.25 W/cm^2^, 3 min, scale bar = 20 µm).

After verifying the light‐controlled NO release of DBTBT‐NO in aqueous solution, we next investigated whether DBTBT‐NO could enter cells and respond to light irradiation within the cellular environment. After incubation with murine breast cancer cells (4T1) for 15 min, distinct red fluorescence from DBTBT‐NO was observed inside the cells via confocal laser scanning microscopy (CLSM) (Figure 3a), indicating efficient cellular uptake. The internalization process was further verified by flow cytometry (Figure S14a). Further co‐localization experiment indicated that DBTBT‐NO mainly accumulated in mitochondria (with a Pearson correlation coefficient of 0.85) rather than lysosome (0.6; Figure 3b). Fluorescent probes DHE, diaminofluorescein‐FM diacetate (DAF‐FM DA), and O56 were used to detect intracellular generation of O_2_ **^·^** ^−^, NO, and ONOO^−^, respectively. As shown in Figure 3c–f, after irradiation of 660 nm laser (0.25 W/cm^2^, 3 min), cells treated with DBTBT‐NO (G5) exhibited bright green fluorescence from DAF‐FM and O56, indicating the generation of NO and ONOO^−^, respectively. These results demonstrated that DBTBT‐NO could effectively enter tumor cells and further respond to deep‐red light. No obvious fluorescence signals from these probes were observed in DBTBT‐NO‐treated cells without light irradiation (G4), suggesting that the NO release from DBTBT‐NO was light‐triggered. Accordingly, spontaneous NO release under dark conditions can be effectively avoided. Compared with cells treated with irradiated DBT (a counterpart lacking the benzisothiadiazole‐based *N*‐nitrosaniline moiety; G3), the red fluorescence intensity of DHE was decreased in G5, which might due to the fast reaction of O_2_ **^·^** ^−^ and NO or inherently less O_2_^–^ generated. The generation of NO and ONOO^−^ was further validated by flow cytometry analysis (Figure S14b,c). Thus, DBTBT‐NO was demonstrated to be a potential therapeutic agent, featuring well ONOO^−^ generation ability triggered by effective release of NO.

### 2.5 Cell Pyroptosis Triggered by Initial NO Release

**FIGURE 4:**
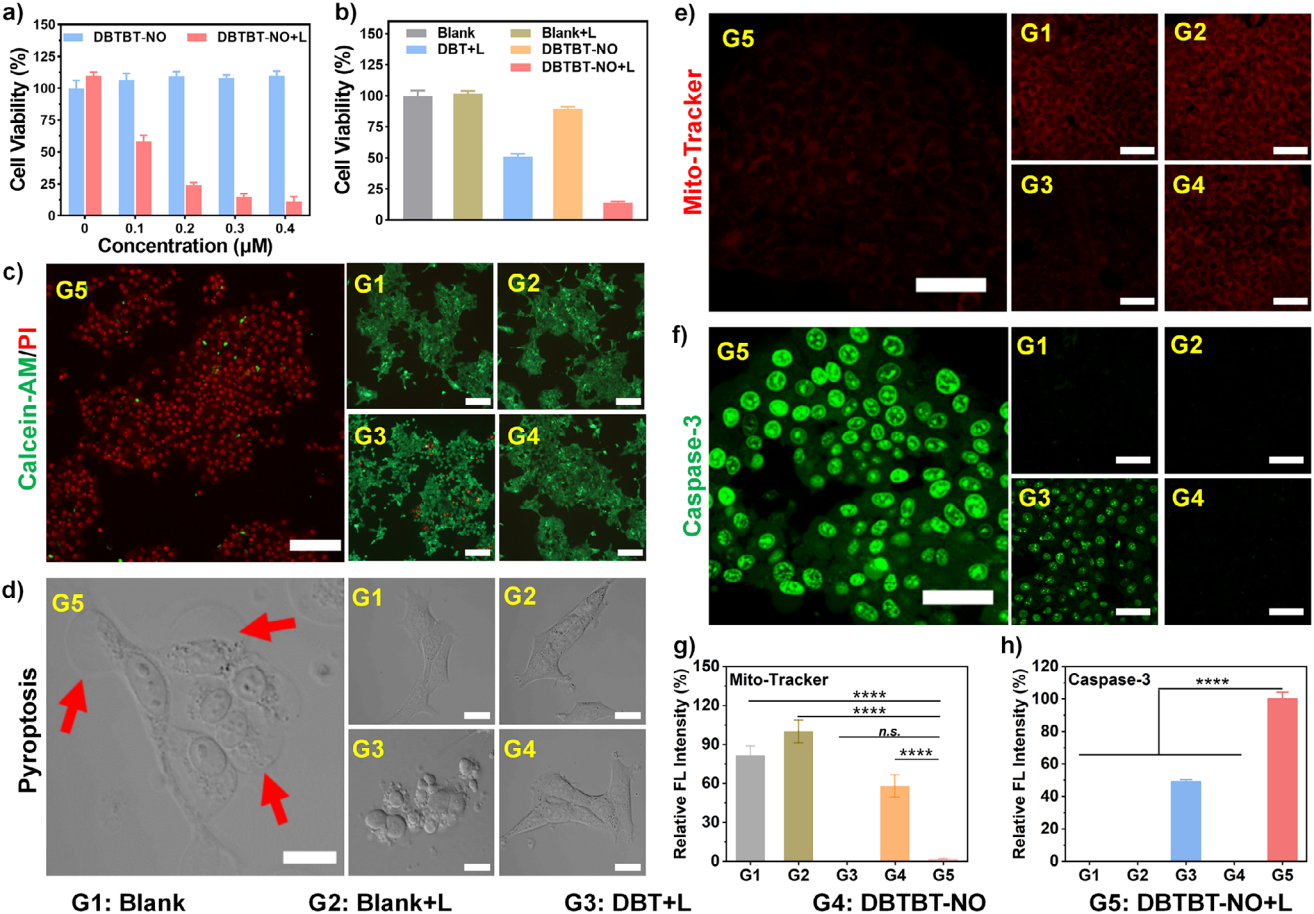
(a) Cell ability of 4T1 cells incubated with different concentrations of DBTBT‐NO in dark or light (660 nm, 0.25 W/cm^2^, 3 min). (b) Cell ability of 4T1 cells after different treatments, the concentration of DBT or DBTBT‐NO was 0.4 µm (660 nm, 0.25 W/cm^2^, 3 min). (c) Live/Dead staining of 4T1 cells by using Calcein‐AM and PI (scale bar = 200 µm) after different treatments in (b). (d) Cell morphological change (bright field) of 4T1 cells after different treatments (660 nm, 0.25 W/cm^2^, 1 min; scale bar = 20 µm). The red arrows indicated the cell bubbling. CLSM images of 4T1 cells, respectively, stained with (e) Mito–Tracker and (f) capase‐3 dyes after different treatments (660 nm, 0.25 W/cm^2^, 3 min; scale bar = 50 µm). (g, h) Quantitative analysis of e, (f). n = 3, mean ± SD. ^****^ *p* < 0.0001.

Since ONOO^−^ was considered to exhibit the strongest toxicity, the cytotoxicity of DBTBT‐NO was next investigated. After irradiation of 660 nm laser at a power density of 0.25 W/cm^2^ for 3 min, DBTBT‐NO exerted a strong inhibitory effect on 4T1 cells even at a low concentration of 0.4 µm (Figure 4a). Whereas it had no impact on cell growth in the absence of light irradiation. At the same low concentration, irradiated DBT only inhibited less than 50% of cell growth (Figure 4b), indicating that ONOO^−^ possessed stronger cell‐killing ability than O_2_ **^·^** ^−^ alone. Live/Dead staining using Calcein‐AM (indicating live cells as green) and PI (indicating dead cells as red) dyes also confirmed the same result (Figure 4c). To clarify the highly efficient killing mechanism, we used CLSM to observe the changes in cell morphology (Figure 4d). Upon only 1 min of light exposure, DBTBT‐NO‐treated cells showed obvious swelling and large bubbles, which is the typical feature of pyroptosis [37, 38]. Cells in other groups showed no significant changes except those in G3. Although the cells in G3 showed many small bubbles, this was more likely caused by apoptosis [39, 40]. These results verified that irradiated DBTBT‐NO is a superior pyroptosis inducer due to its unique NO release mechanism. Additionally, the photo‐induced pyroptosis efficiency in this work was higher than that reported in the current literature (Figure S15).

The cross‐linked signal network in cells may induce concurrence of varied cell death pathways [41, 42]. Caspase‐3 has long been considered as a indicator of apoptosis. Nonetheless, caspase‐3 can cleave gasdermin E (GSDME) to generate its N‐terminal domain (GSDME‐N), inducing the pyroptosis that is different from caspase‐1/gasdermin‐D pathway [43, 44]. Since DBTBT‐NO was mainly located in cellular mitochondria, the damage to mitochondria and the expression of caspase 3 were next investigated. As shown in Figure 4e,g, the red fluorescence intensity in G5 was remarkably decreased and extremely weak compared with G1, G2, and G4, indicating that irradiated DBTBT‐NO induced a reduction in mitochondrial membrane potential. Meanwhile, the high intensity of green fluorescence was observed in G5, indicating the abundant expression of caspase 3 (Figure 4f,h). Since apoptosis is accompanied by mitochondrial dysfunction and caspase 3 expression, the cells in G3 also exhibited similar signals but a lower expression level of capase 3 than those in G5. The pyroptosis process is reported to promote immunogenic cell death (ICD) and enhance immune response [40]. As expected, the calreticulin (CRT; a characteristic marker of ICD) expression in G5 was the highest compared with that in other groups (Figure S16), indicating the activation of strong ICD [45]. Based on the above‐mentioned capabilities, DBTBT‐NO exhibited obvious advantages over Ce6 (a commercial photosensitizer) in cell killing at low concentrations (Figure S17).

Besides 4T1 cells, murine melanoma cells (B16‐F10) and human cervical cancer cells (HeLa) were also employed to investigate the efficacy of DBTBT‐NO in triggering pyroptosis and cell death of tumor cells. As shown in Figure S18, irradiated DBTBT‐NO could also efficiently kill two another tumor cells and trigger obvious pyroptosis. Considering that hypoxic regions are prevalent in the microenvironment of solid tumors, the cell killing efficiency of irradiated DBTBT‐NO under hypoxic conditions was investigated (Figure S19). Compared to aerobic conditions, the killing efficiency of DBTBT‐NO was slightly decreased under hypoxic conditions, suggesting good tolerance to low‐oxygen environments. Although DBTBT‐NO showed high light cytotoxicity against tumor cells, it had no influence on the growth of normal cells (L929) without light irradiation (Figure S20), indicating the specific killing effect and good cytocompatibility. Additionally, the hemocompatibility of DBTBT‐NO was further investigated, and the result is shown in Figure S21. Even at 8 times the cell‐killing concentration (3.2 µm), DBTBT‐NO did not trigger an obvious hemolytic reaction. All the above results demonstrated that irradiated DBTBT‐NO holds great potential in effective and safe antitumor therapy.

### 2.6 Antitumor Effect In Vivo

**FIGURE 5:**
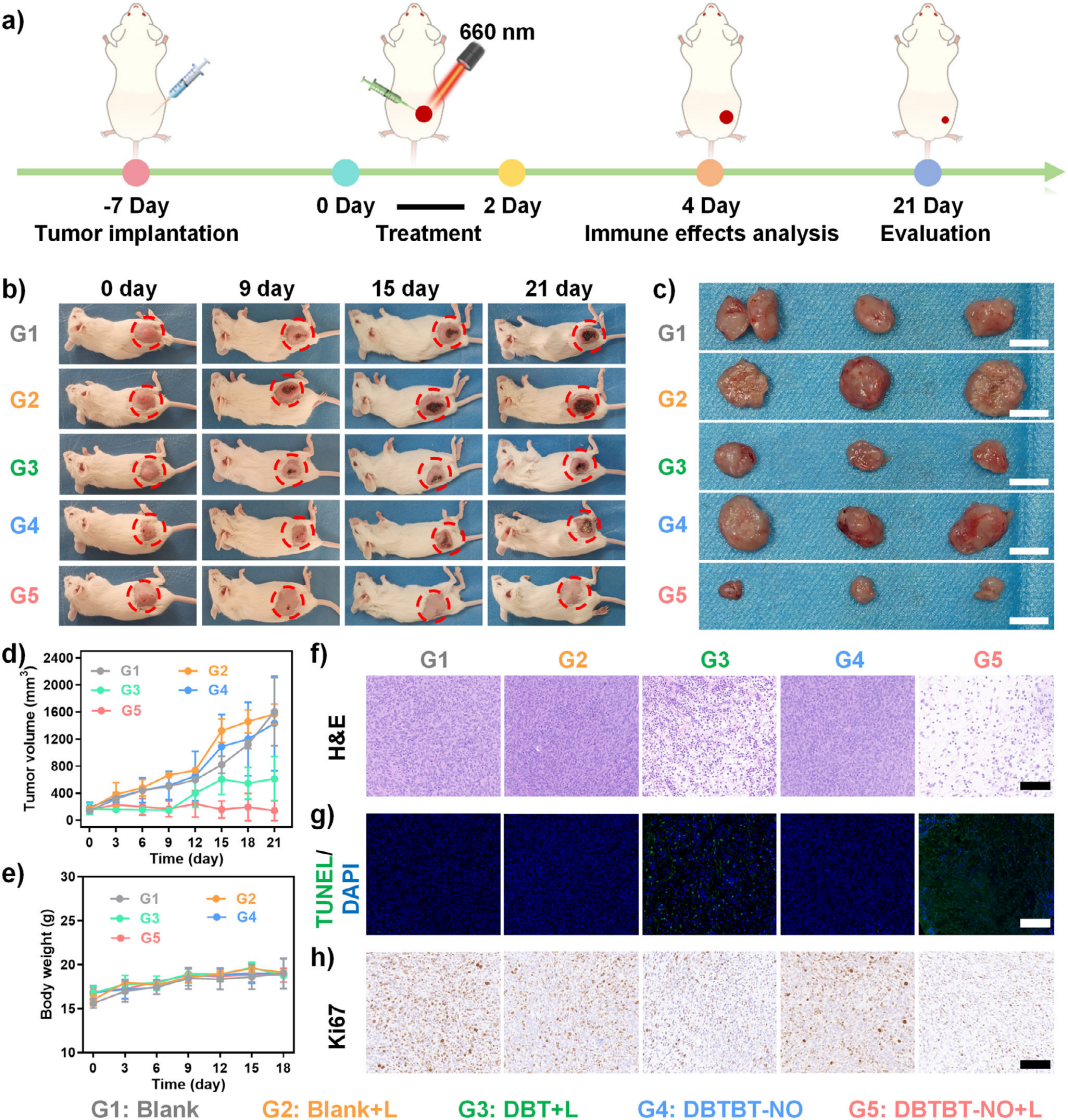
(a) Schematic illustration of the treatment schedule. (b) Representative photographs of mice after different treatments on different days during treatment. (c) Representative ex vivo tumor images in different groups after treatment completed (scale bar = 1 cm). Growth curves of (d) tumor and (e) body weight of mice in different groups. (f) H&E staining, (g) immunofluorescent staining of TUNEL, and (h) immunohistochemical staining of Ki67 images of tumor tissues in different groups, scale bar = 100 µm. (n = 3, mean ± SD).

Encouraged by the highly efficient cell‐killing ability of DBTBT‐NO, a 4T1 tumor‐bearing mouse model was established to evaluate its antitumor activity in vivo. The treatment schedule was shown in Figure 5a. When the tumor volume reached 100 mm^3^, all mice were randomly divided into 5 groups: Blank (G1), Blank+L (G2), DBT+L (G3), DBTBT‐NO (G4), and DBTBT‐NO+L (G5). After injecting and light irradiation (660 nm, 0.25 W/cm^2^, 3 min) for 3 times, the tumor volume and body weights of mice were measured every 3 days until the treatment was finished on day 21. Compared with other groups, the tumor growth in G5 was significantly inhibited (Figure 5b–d). Tumor tissues were excised to perform hematoxylin and eosin (H&E) and terminal deoxynucleotidyl transferase dUTP nick end labeling (TUNEL) staining for further investigation of antitumor effect. In contrast to the intact cellular morphology observed in other groups, tissues in G5 exhibited extensive nuclear pyknosis and loss (Figure 5f), indicating severe cell damage. TUNEL/DAPI staining revealed the strongest green fluorescence in G5, corresponding to the highest level of cell apoptosis (Figure 5g). Additionally, the lowest expression of Ki‐67 in G5 indicated that tumor cells lost their proliferative ability (Figure 5h). During the treatment period, no significant changes in body weight were observed in any of the mice, including those in G5 (Figure 5e). Additionally, routine blood analysis and H&E staining of major organs of mice demonstrated no obvious abnormalities across all groups, suggesting that irradiated DBTBT‐NO did not cause additional toxicity to the mice during treatment (Figures S22 and S23). Collectively, these results indicated that irradiated DBTBT‐NO could release NO to induce ONOO^−^‐mediated tumor cell death in vivo with well biocompatibility.

### 2.7 Anti‐Tumor Immune Response In Vivo

**FIGURE 6:**
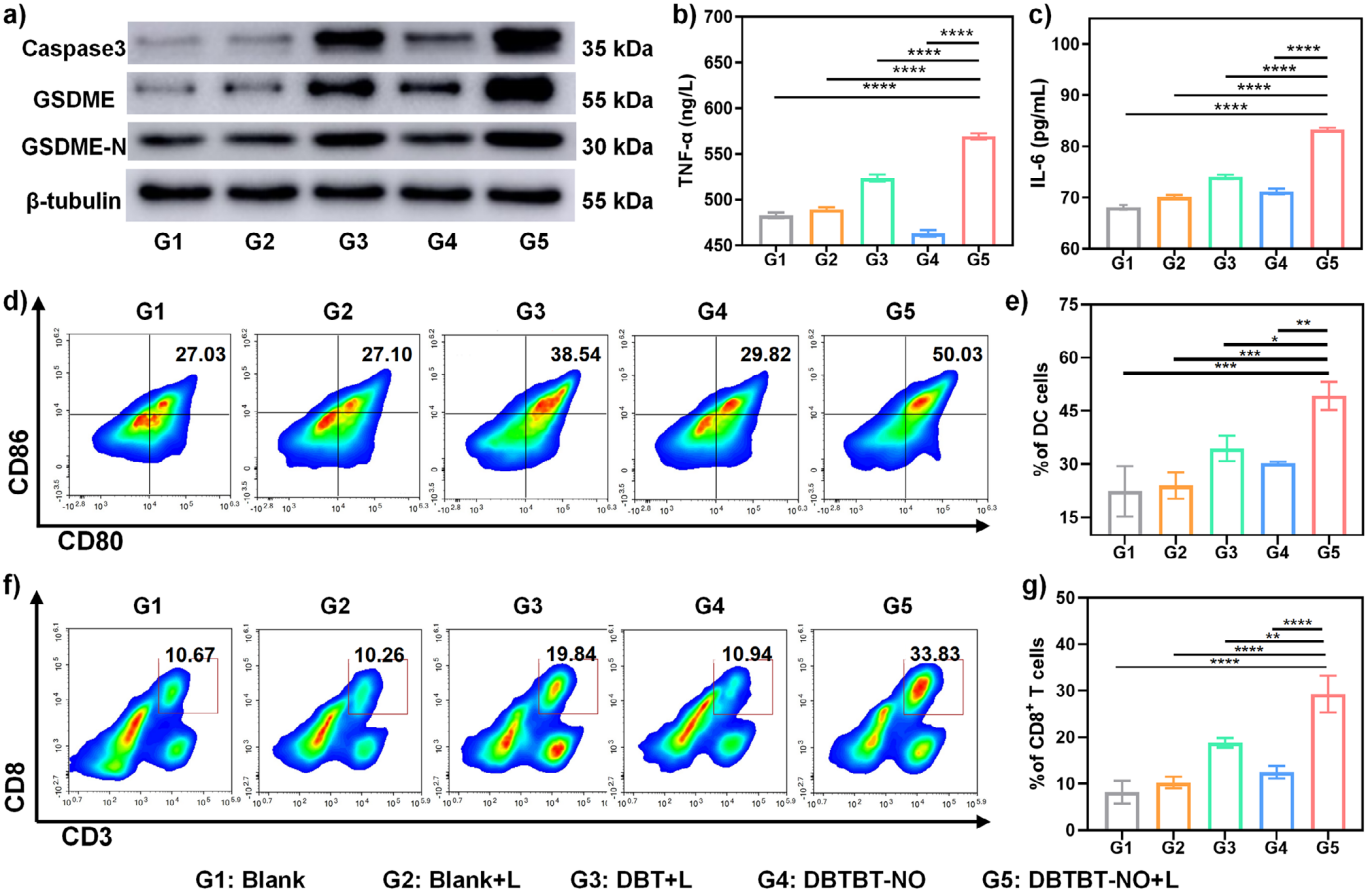
(a) Expression levels of pyroptosis‐associated proteins in tumors after different treatments. (b) TNF‐α and (c) IL‐6 levels in the serum of mice in different groups. (d) FCM analysis of mature DCs in different groups and corresponding (e) quantitative analysis results. (f) FCM analysis of CD8^+^ T cells gating on CD3 cells within tumors in different groups and corresponding (g) quantitative analysis result. (n = 3, mean ± SD, ^*^ *p* <0.05, ^**^ *p* < 0.01, ^***^ *p* < 0.001, ^****^ *p* < 0.0001).

To further explore the antitumor mechanism, western blot analysis was performed to detect the expression levels of pyroptosis‐related proteins in tumor tissues on day 4. As shown in Figure 6a, the expression levels of caspase‐3, GSDME, and GSDME‐N were the highest in G5, confirming that irradiated DBTBT‐NO activated pyroptosis in mice. Since pyroptosis is a lytic programmed cell death, the pores formed in the cell membrane can release intracellular contents and pro‐inflammatory cytokines to induce a strong immune response [46, 47, 48]. Then, an enzyme‐linked immunosorbent assay (ELISA) was performed to detect the levels of pro‐inflammatory cytokines in serum, such as tumor necrosis factor α (TNF‐α), interleukin 6 (IL‐6), and interferon‐γ (IFN‐γ). The results showed that the expression levels of these pro‐inflammatory cytokines were the highest in the G5 group, further demonstrating the occurrence of pyroptosis and the promotional effect of irradiated DBTBT‐NO on pro‐inflammatory cytokines release (Figure 6b,c, Figure S24). Simultaneously, the proportion of mature DCs related to antigen presentation was analyzed by flow cytometry. The results showed that the percentage of mature DCs in G5 (50.03%) was significantly higher than that in groups G1 (27.03%), G2 (27.10%), G3 (38.54%), and G4 (29.82%) (Figure 6d,e), verifying the promotion effect of irradiated DBTBT‐NO on DCs maturation by triggering pyroptosis. Since CD8^+^ T cells can be activated by mature DCs and play an important role in anti‐tumor immune response by recognizing and killing tumor cells [49, 50], the production efficiency of CD8^+^ T cells was then evaluated by flow cytometry (Figure 6f,g). As expected, the proportion of CD8^+^ T cells in G5 (33.83%) was also the highest among all groups (G1: 10.67%, G2: 10.26%, G3: 19.84%, and G4: 10.94%), indicating the successful infiltration of CD8^+^ T cells in the tumor tissues of G5. The strong antitumor response not only enhances the killing efficacy of primary tumors but also effectively suppresses tumor metastasis [51]. Hence, metastatic nodules in the lungs of mice after various treatments were counted, and the results demonstrated that irradiated DBTBT‐NO inhibited pulmonary metastasis in mice (Figure S25). All these results reveal that red light‐triggered NO release successfully induces the pyroptosis of tumor cells and activates a potent immune response, which inhibits in vivo tumor metastasis through a cascade reaction to generate ONOO^−^. Notably, these achievements were accomplished by using a single synthetic molecule, without the assistance of any other auxiliary agent, such as immunoadjuvants.

## 3 Conclusions

In summary, we report a novel strategy to achieve deep‐red light‐activated NO release from benzisothiadiazole‐based *N*‐nitrosanilines without modification of nitro moieties for the first time. By covalently conjugating a benzisothiadiazole‐based *N*‐nitrosaniline group with Nile blue derivatives directly via flexible spacers, N‐N bond cleavage can be activated through light‐induced intramolecular electron transfer. Through rational molecular design, the representative derivative DBTBT‐NO was synthesized and demonstrated to generate massive ONOO^−^ via the rapid reaction of NO and O_2_ **^·^** ^−^ during the photolysis process. Additionally, DBTBT‐NO specifically targets tumor cell mitochondria and triggers robust pyroptosis with a strong immune response at a low light dose of 15 J/cm^2^ and low concentration of 0.4 µm, which is lower than those reported in the current literature. The high pyroptosis efficiency mediated by DBTBT‐NO was verified to enhance antitumor therapy in tumor‐bearing mice. This work provides a feasible strategy for fabricating high‐efficiency ONOO^−^ generators based on deep‐red light‐triggered N‐N bond cleavage, advancing the development of photoexcitation‐mediated electron transfer theory and photoactivatable antitumor immunotherapy.

## 4 Experimental Section/Methods

### 4.1 Synthesis of BT‐NH_2_

4‐Bromo‐2,1,3‐benzothiadiazole (500 mg, 2.3 mmol), 1,3‐propanediamine (970 µL, 11.6 mmol), copper powder (7 mg, 0.12 mmol), and water (800 µL) were added sequentially to a pressure tube (15 mL), followed by reaction at 100°C for 3 h. Then the mixture was cooled to room temperature and extracted three times with dichloromethane (30 mL). After dried over anhydrous sodium sulfate, the solvent was removed under reduced pressure. The residue was purified by column chromatography (methanol/dichloromethane = 1:10, v/v, containing 1% triethylamine) to obtain BT‐NH_2_ as yellow oily liquid (367 mg, yield 74%).^1^H NMR (600 MHz, DMSO‐*d_6_*, δ): 7.49‐7.41 (m, 1H), 7.11 (d, 1H), 6.85 (t, 1H), 6.37 (d, 1H), 3.32 (q, 2H), 2.68 (t, 4H), 1.73 (p, 2H); HRMS (ESI) *m*/*z*: [M + H]^+^ calcd for C_9_H_12_N_4_S, 209.0855; found, 209.0850.

### 4.2 Synthesis of DBTBT‐H

DBT (200 mg, 0.43 mmol), BT‐NH_2_ (135 mg, 0.65 mmol), N, N‐dimethylformamide (DMF, 4 mL), triethylamine (179 µL), and 406 mg HATU (1.07 mmol) were added to a round‐bottom flask (25 mL) and reacted for 1 h. Then water was added to obtain a precipitate. The crude product was purified by column chromatography (methanol/dichloromethane = 1:30, v/v) to obtain DBTBT‐H as black solid (150 mg, yield 54%).^1^H NMR (400 MHz, DMSO‐*d_6_*, δ): 8.92 (d, 1H), 8.44 (d, 1H), 8.08 (t, 1H), 7.91 (dd, 2H), 7.84 (t, 1H), 7.47 (s, 1H), 7.36 (s, 1H), 7.33 (d, 1H), 7.30 (d, 1H), 7.02 (d, 1H), 6.61 (t, 1H), 6.29 (d, 1H), 3.65 (t, 4H), 3.24 (dq, 4H), 3.11 (q, 2H), 2.36 (t, 2H), 2.03 (q, 2H), 1.78 (p, 2H), 1.24 (t, 6H); ^13^C NMR (151 MHz, CD_3_OD, δ): 173.96, 155.80, 153.26, 151.43, 148.73, 140.21, 137.20, 137.19, 134.02, 133.66, 132.99, 132.97, 132.03, 130.89, 129.45, 129.22, 125.01, 124.28, 122.96, 122.09, 121.15, 117.45, 104.77, 101.80, 45.43, 43.83, 43.36, 36.67, 32.58, 26.38, 23.60, 11.61; HRMS (ESI) *m*/*z*: [M + H]^+^ calcd for C_33_H_36_N_7_OS_2_, 610.2417; found, 610.2419.

### 4.3 Synthesis of DBTBT‐NO

DBTBT‐H (150 mg, 0.23 mmol), acetic acid (CH_3_COOH, 4 mL), and tetrahydrofuran (THF, 6 mL) were added to a round‐bottom flask (50 mL). After cooling in an ice‐water bath, sodium nitrite (79 mg, 1.15 mmol) was added to react for 2 h. Then, a saturated sodium bicarbonate solution was added to neutralize the excess acetic acid. The aqueous layer was extracted three times with dichloromethane (30 mL), then dried over anhydrous sodium sulfate. After removing the solvent, the residue was purified by column chromatography (methanol/dichloromethane = 1:30, v/v) to obtain DBTBT‐NO as a black solid (87 mg, yield 56%). ^1^H NMR (400 MHz, DMSO‐*d_6_*, δ): 9.02–8.96 (m, 1H), 8.47 (t, 1H), 8.15 (dd, 1H), 8.01 (dd, 1H), 7.94 (t, 2H), 7.90–7.86 (m, 1H), 7.86–7.82 (m, 1H), 7.82–7.77 (m, 1H), 7.52 (s, 1H), 7.41 (dd, 2H), 4.33–4.22 (m, 2H), 3.67 (q, 6H), 3.05 (q, 2H), 2.24 (t, 2H), 2.00–1.90 (m, 2H), 1.64 (p, 2H), 1.24 (t, 6H); ^13^C NMR (151 MHz, CD_3_OD, δ): 174.17, 163.46, 155.53, 153.10, 151.30, 147.53, 140.23, 137.09, 133.92, 133.57, 132.83, 131.91, 131.71, 130.78, 129.37, 124.97, 124.19, 122.04, 117.33, 106.61, 104.56, 101.58, 100.44, 45.40, 43.89, 40.40, 36.95, 35.53, 32.70, 30.24, 28.02, 26.70, 23.46, 13.03, 11.66; HRMS (ESI) *m*/*z*: [M + H]^+^ calcd for C_33_H_35_N_8_O_2_S_2_, 639.2319; found, 639.2319.

### 4.4 Detection of O_2_^−^

DHE was used as an indicator to detect O_2_ **^·^** ^−^ generated in different solutions. The DHE test solution was prepared by adding 200 µL of DHE (1 mg/mL in DMSO) to PBS solution containing Ct DNA (2.0 mg). Then, the solutions of DBTBT‐NO (5.0 µm) were irradiated with a 660 nm laser (1.0 W/cm^2^) for 5 min. The fluorescence intensity at 590 nm (λ_ex_: 540 nm) was recorded every minute.

### 4.5 Detection of NO

The concentration of released NO was measured using the Griess reagent. Specifically, NO reacts with water to form nitrate or nitrite, which then reacts with the Griess reagent to produce an azo dye with a strong absorption peak at 540 nm. Quantitative detection was achieved by measuring the absorbance value at 540 nm. Solutions of DBTBT‐NO (5.0 µm) were irradiated with a 660 nm laser (1.0 W/cm^2^) for 5 min to perform the NO measurement.

### 4.6 Detection of ONOO^−^

L‐tyrosine was used as a probe to detect ONOO^−^ generation. L‐tyrosine (0.5 mM) was dissolved in PBS buffer (0.1 m, pH 8.2) containing NaHCO_3_ (15.0 mM) to serve as the control solution. Solutions of L‐tyrosine and DBTBT‐NO (5.0 µm) were irradiated with a 660 nm laser (1.0 W/cm^2^) for 5 min to study ONOO^−^ generation.

### 4.7 MD Simulations

The all‐atom classical MD simulation was carried out with the Amber 24 package [52]. Force field parameters for the DBTBT‐NO molecule were generated at the B3LYP/6‐31+g* level by processes of geometry optimization and parameter calculation in the Gaussian 09 package [53]. The TIP3P model was selected for the water molecule. During MD simulations, energy minimizations were performed using a combination of steepest descent and conjugate gradient algorithms. All production simulations were performed in the NPT ensemble. The periodic boundary conditions were used. All bonds involving hydrogen were controlled by the SHAKE algorithm [54]. The temperature was kept constant using a Langevin thermostat at 300 K with a collision frequency of 2 ps^−1^. The pressure was controlled by the Berendsen barostat at 1 atm with a pressure relaxation time of 2 ps. The electrostatic term was computed using the Particle‐Mesh‐Ewald summation method [55]. The cutoff algorithm was set as 10 Å, and the time step was set as 1 fs.

In the initial structure modeling, the cubic box was set to be 118 Å × 125 Å ×125 Å for each system (the number of solvent molecules was 30 for DBTBT‐NO). Finally, in the current study, a total 600 ns trajectory was collected for each system. After the equilibration checking, only the final converged 100 ns trajectory was used for further analyses. In addition, structural changes of molecular were exhibited using the visual molecular dynamics (VMD) software [56].

### 4.8 Density Functional Theory (DFT) Calculation

All DFT calculations, including coordination scan searches, were conducted using the Gaussian 09 package at the B3LYP/6‐31 g level. During the coordinate scan, selected internal coordinates were frozen while the NO remaining degrees of freedom were fully optimized, allowing the potential energy surface to be explored along the designated reaction coordinate. After the scanning, the energy was calculated based on the polarizable continuum water model at the B3LYP/6‐31+g* level. Moreover, we calculated the electron‐hole distribution. The related properties were visualized by using Multiwfn 3.8 [57].

### 4.9 Cellular Uptake and Location of DBTBT‐NO

4T1 cells were used to study the cellular uptake capacity of DBTBT‐NO. Cells were seeded in confocal imaging dishes at a density of 8 × 10^3^ cells/well, and DBTBT‐NO (0.4 µm) was added for incubation at 37°C. The intracellular fluorescence of DBTBT‐NO at different time points was observed using CLSM.

To determine the subcellular localization of DBTBT‐NO, 4T1 cells were seeded into confocal imaging dishes at a density of 2 × 10^5^ cells/well, followed by incubation at 37°C for 24 h. After removing the old medium, DBTBT‐NO (0.4 µm) was added to the dishes and incubated for 30 min. After washing with PBS, 1.0 mL of Mito‐Tracker Green (100 nM) and Lyso–Tracker Green (100 nM) were respectively added for another incubation for 30 min at 37°C. After changing the medium, CLSM imaging was performed under the following conditions: DBTBT‐NO was excited at 633 nm, and fluorescence was collected at 710 nm; Lyso‐Tracker Green was excited at 504 nm, and fluorescence was collected at 511 nm; Mito‐Tracker Green was excited at 490 nm, and fluorescence was collected at 516 nm.

### 4.10 Detection of Intracellular O_2_ **^·^** ^−^

4T1 cells were seeded into confocal imaging dishes at a density of 2 × 10^5^ cells/well. The cells were divided into five groups: Blank group, Blank+L group, DBT+L group, DBTBT‐NO group, and DBTBT‐NO+L group (L: laser irradiation). The concentrations of DBT and DBTBT‐NO were both 0.4 µm. All groups were incubated for 30 min. After removing the old medium, all cells were incubated with 1.0 mL of DHE (10.0 µm) for 30 min. Then, cells in the laser groups were irradiated with a 660 nm laser (0.25 W/cm^2^) for 3 min. Finally, all the cells were washed with PBS for three times to perform CLSM imaging with an excitation wavelength of 535 nm and fluorescence collection between 590–610 nm.

### 4.11 Detection of Intracellular NO

4T1 cells were seeded into confocal imaging dishes at a density of 2 × 10^5^ cells/well and divided into five groups as above‐mentioned (concentrations of DBT and DBTBT‐NO: 0.4 µm) for incubation of 30 min at 37°C. After removing the old medium, all cells were incubated with 1.0 mL of the NO fluorescence probe DAF‐FM DA (2.0 µm) for 30 min. Cells in the laser groups were irradiated with a 660 nm laser (0.25 W /cm^2^) for 3 min. Finally, all the cells were washed with PBS for three times to perform CLSM imaging with an excitation wavelength of 495 nm and fluorescence collection at 515 nm.

### 4.12 Detection of Intracellular ONOO^−^

4T1 cells were seeded into confocal imaging dishes at a density of 2 × 10^5^ cells/well and divided into five groups as above‐mentioned (concentrations of DBT and DBTBT‐NO: 0.4 µm) for incubation of 30 min at 37°C. After removing the old medium, all cells were incubated with 1.0 mL of the ONOO^−^ fluorescence probe O56 (2.0 µm) for 30 min. Cells in the laser groups were irradiated with a 660 nm laser (0.25 W/cm^2^) for 3 min. Finally, all the cells were washed with PBS for three times to perform CLSM imaging with excitation wavelength of 488 nm and fluorescence collection at 530 nm.

### 4.13 Detection of Mitochondrial Membrane Potential and Caspase‐3 Release

A live‐cell caspase‐3 activity and mitochondrial membrane potential detection kit was used to measure caspase‐3 expression and mitochondrial membrane potential in 4T1 cells. Cells were seeded into confocal imaging dishes at a density of 2 × 10^5^ cells/well and divided into five groups as above‐mentioned (concentrations of DBT and DBTBT‐NO: 0.4 µm) for incubation of 30 min at 37°C. Cells in the laser groups were exposed to a 660 nm laser (0.25 W/cm^2^) for 3 min. Then, 200 µL of detection solution was added to each well for another incubation of 30 min. After washing with PBS, cells were performed CLSM imaging under the following conditions: GreenNuc‐DNA was excited at 500 nm, and fluorescence was collected at 530 nm (green fluorescence); Mito–Tracker Deep Red 633 was excited at 622 nm, and fluorescence was collected at 648 nm (red fluorescence).

### 4.14 Cell Activity Measurement

4T1 cells were incubated in 96‐well plate (8 × 10^3^ cells/well) for 24 h. After that, different concentrations of DBTBT‐NO were added and incubated with cells for 4 h. Cells in the laser groups were irradiated with a 660 nm laser (0.25 W/cm^2^) for 3 min, followed by incubation for 4 h. Then, the old medium in each well was replaced with 100 µL of medium containing MTT (0.5 mg/mL). After incubation for 4 h, the supernatant was carefully removed, and 100 µL of DMSO was added to each well. The absorbance value of each well at 490 nm was measured and recorded using a Bio‐Rad microplate reader.

### 4.15 Live/Dead Staining of Cells

4T1 cells were seeded into confocal imaging dishes at a density of 5 × 10^3^ cells/well. The cells were divided into five groups (Blank group, Blank+L group, DBT+L group, DBTBT‐NO group, and DBTBT‐NO+L group; concentrations of DBT and DBTBT‐NO: 0.4 µm) and incubated at 37°C for 30 min. Cells in the laser groups were exposed to a 660 nm laser (0.25 W/cm^2^) for 3 min, followed by incubation for 4 h. After washing with PBS, cells were stained with Calcein‐AM/PI for 30 min. Then the cells were washed three times with PBS to perform fluorescent imaging by a fluorescence microscope (Zeiss).

### 4.16 ICD Assay

4T1 cells were seeded into confocal imaging dishes at a density of 5 × 10^3^ cells/well. The cells were divided into five groups as above‐mentioned (concentrations of DBT and DBTBT‐NO: 0.4 µm) and incubated at 37°C for 30 min. Cells in the laser groups were exposed to a 660 nm laser (0.25 W/cm^2^) for 3 min, followed by incubation for 4 h. After washing with PBS, cells were then incubated with anti‐CALR antibody (1:200 dilution) at 4°C for 2 h. Then the cells were washed and incubated with Alexa Fluor 488‐conjugated secondary antibody (1:200 dilution) at 4°C for another 1 h. After stained with DAPI for 10 min, the cells were washed with PBS to perform CLSM imaging: CALR was excited at 488 nm with fluorescence collected at 530 nm, and DAPI was excited at 405 nm with fluorescence collected at 460 nm. The cell supernatant was collected for the detection of HMGB1 content.

### 4.17 In Vivo Antitumor Therapy

BALB/c mice (female, 6–8 weeks) were provided by the Tianjin Yishengyuan Gene Technology Co., Ltd. And the animal experiments were authorized by the Tianjin Yishengyuan Gene Technology Co., Ltd. Balb/c mice were subcutaneously inoculated with 4T1 cells (1 × 10^6^ cells/100 µL PBS) to establish tumor models. When the tumor volume reached 100 mm^3^, the mice were divided into five groups: Blank group, Blank+L group (0.25 W/cm^2^, 3 min), DBT+L group (0.25 W/cm^2^, 3 min), DBTBT‐NO group, and DBTBT‐NO+L group (0.25 W/cm^2^, 3 min). The concentrations of DBT and DBTBT‐NO were both 0.4 µm. The body weight of the mice was recorded every 3 days, and the tumor volume was monitored daily using a vernier caliper throughout the experiment. The tumor volume was calculated using the formula: V = W^2^ × L/2 (where L is the longest diameter of the tumor, and W is the shortest diameter of the tumor).

### 4.18 In Vivo Antitumor Immune Activation

On the fourth day after the completion of treatment, a part of the mice in each group were sacrificed. Blood samples were collected to evaluate blood biochemical indicators, and an ELISA was performed to detect the levels of IL‐6, TNF‐α, and IFN‐γ. Tumor tissues and major organs were excised to conduct H&E staining and immunohistochemical staining on different biomarkers using corresponding assay kits and antibodies.

## Funding

National Natural Science Foundation of China (Nos. 22375119, 22177065, and U21A6004), Fundamental Research Program of Shanxi Province (Nos. 202403021224002)

## Conflicts of Interest

The authors declare no conflicts of interest.

## Supporting information




**Supporting File**: advs75841‐sup‐0001‐SuppMat.doc.

## Data Availability

The data that support the findings of this study are available in the supplementary material of this article.
