# Supplementary material for: Light‐Activatable Nitric Oxide Release via Intramolecular Electron Transfer for Tumor Pyroptosis Induction
Source: Adv Sci (Weinh). 2026 May 25:e22486. Online ahead of print. doi: 10.1002/advs.202522486 (PMC13335896; doi:10.1002/advs.202522486)
Supplement: Supplementary file 1 — Supporting File: advs75841‐sup‐0001‐SuppMat.doc. [file ADVS-9999-e22486-s001.doc]

Supporting Information

Light-Activatable Nitric Oxide Release via intramolecular electron transfer for Tumor Pyroptosis Induction

Chuangxin Zhang,^a^ Ruipeng Li,^a^ Yunxia Wang,*^a^ Feng Liu,*^a,b^ and Liheng Feng*^a^

C. Zhang, R. Li, Y. Wang, F. Liu, L. Feng

School of Chemistry and Chemical Engineering, Shanxi University, Taiyuan 030006, China.

E-mail: wangyunxia@sxu.edu.cn; feng.liugift25@sjtu.edu.cn; lhfeng@sxu.edu.cn

F. Liu

Future Organic Optoelectronics Research Center, Global Institute of Future Technology, Shanghai Jiao Tong University, Shanghai 200240, China

E-mail: feng.liugift25@sjtu.edu.cn

**1. Experimental Sections**

*Materials and apparatus*: All chemicals were purchased from commercial sources and used without further treatment. 2,7-dichlorofluorescein diacetate (DCFH-DA) were obtained from Sigma-Aldrich. 3-Amino-4-aminomethyl-2′,7′-fluorescein diacetate (DAF-FM DA) was purchased from Beyotime Biotech Inc. ONOO^−^ fluorescence probe (BB-46065) was purchased from Shanghai Bestbio Biotechnology Co., Ltd. Di-hydroethidium (DHE), anti-CALR antibody and Alexa Fluor 488 conjugated secondary anti-rabbit antibody were obtained from Shanghai Maokang Biotechnology Co., Ltd. Calcein-AM, PI were obtained from Solarbio Biotechnology Co., Ltd. ELISA kits for HMGB1 were obtained from swamp Biotechnology Co., Ltd. Lyso-Tracker Green, Mito-Tracker Green, Total Glutathione Assay Kit was purchased from Beyotime Biotechnology Co., Ltd. 4T1 cell lines were purchased from Wuhan Procell Life Technology Co., Ltd. UV-Vis absorption spectra were recorded on a UV spectrophotometer (Hitachi UH5300, Japan). Fluorescence emission spectra were measured on a fluorescence spectrophotometer (Hitachi F-4600, Japan) with a Xenon lamp as the excitation source. Sizes and zeta potentials were measured by a Zeta potentiometer (Malvern ZetaSizer Nano ZS90, America). The morphology of nanoparticles was observed by transmission electron microscope (TEM, JEOL, JEM-F200, Japan). Cell viability was measured by a microplate reader (Bio-Rad, England). Fluorescence images were captured by a confocal laser scanning microscopy (CLSM, Zeiss LSM 880, Germany).

*Synthesis of DBT-H*: DBT (250 mg, 0.6 mmol), propylamine (50 mg, 0.9 mmol), N, N-dimethylformamide (DMF, 5 mL), triethylamine (830 μL, 6.0 mmol), and HATU (570 mg, 1.5 mmol) were added to a round-bottom flask (25 mL) and reacted for 3 h. Then water was added to obtain precipitate. The crude product was purified by column chromatography (methanol/dichloromethane = 1:50, v/v) to obtain DBT-H as black solid (128 mg, yield 46%).^1^H NMR (600 MHz, DMSO-*d_6_*, δ): 9.95 (s, 1H), 8.99 (s, 1H), 8.48 (d, J = 7.5 Hz, 1H), 8.04 – 7.98 (m, 1H), 7.93 (s, 1H), 7.88 (t, J = 7.2 Hz, 1H), 7.58 (s, 1H), 7.45 (s, 1H), 7.41 (d, J = 9.1 Hz, 1H), 3.67 (d, J = 6.6 Hz, 6H), 3.03 (q, J = 5.9 Hz, 2H), 2.31 (t, J = 6.5 Hz, 2H), 2.00 – 1.96 (m, 2H), 1.40 (q, J = 7.2 Hz, 2H), 1.24 (t, J = 7.0 Hz, 6H), 0.83 (t, J = 7.2 Hz, 3H); ^13^C NMR (101 MHz, DMSO-*d_6_* δ): 172.15, 153.53, 151.30, 137.30, 134.13, 133.65, 132.59, 131.64, 130.10, 130.01, 125.19, 123.58, 117.82, 105.91, 103.55, 45.60, 40.87, 32.70, 24.50, 22.84, 13.13, 11.91; HRMS (ESI) *m*/*z*: [M]^+^ calcd for C_27_H_33_N_4_OS, 461.2370; found, 461.2364.

*Synthesis of DBT-NO*: DB-H (128 mg, 0.24 mmol), acetic acid (CH₃COOH, 4 mL), and tetrahydrofuran (THF, 4 mL) were added to a round-bottom flask (50 mL). After cooled in ice-water bath, sodium nitrite was added to neutralize the excess acetic acid. The aqueous layer was extracted three times with dichloromethane (30 mL), then dried over anhydrous sodium sulfate. After removed solvent, the residue was purified by column chromatography (methanol/dichloromethane = 1:30, v/v) to obtain DBT-NO as black solid (70 mg, yield 60%). ^1^H NMR (600 MHz, CD_3_OD, δ): 8.87 (d, J = 8.0 Hz, 1H), 8.17 (d, J = 7.8 Hz, 1H), 7.86 (d, J = 9.2 Hz, 1H), 7.79 (d, J = 7.7 Hz, 1H), 7.73 (d, J = 7.2 Hz, 1H), 7.29 (d, J = 9.2 Hz, 1H), 7.15 (s, 1H), 7.09 (s, 1H), 3.67–3.57 (m, 6H), 3.16 (t, J = 6.5 Hz, 2H), 2.44 (s, 2H), 2.09 (s, 2H), 1.52 (q, J = 6.7 Hz, 2H), 1.31 (d, J = 6.3 Hz, 6H), 0.92 (t, J = 7.1 Hz, 3H); ^13^C NMR (101 MHz, CD_3_OD, δ): 173.85, 152.98, 151.24, 139.77, 137.03, 133.69, 133.27, 132.64, 131.77, 130.74, 129.31, 124.85, 124.10, 121.99, 117.26, 104.62, 101.71, 45.44, 43.96, 41.00, 32.69, 23.73, 22.25, 11.71, 10.38; HRMS (ESI) *m*/*z*: [M]^+^ calcd for C_27_H_32_N_5_O_2_S, 490.2271; found, 490.2243.

*Statistical Analysis*: All experimental data were presented as mean value ± standard deviation (SD). All statistical analyses were performed using Prism software (GraphPad Prism version 8.0.2; www.graphpad. com). Statistical differences between groups were performed by a one-way analysis of variance (ANOVA). In all cases, significance was defined as *P <* 0.05. **P <* 0.05, ***P <* 0.01, ****P <* 0.001 and *****P <* 0.001 represent different significant differences. For all experiments, n = 3 independent experiments.

**2. Supporting Figures**


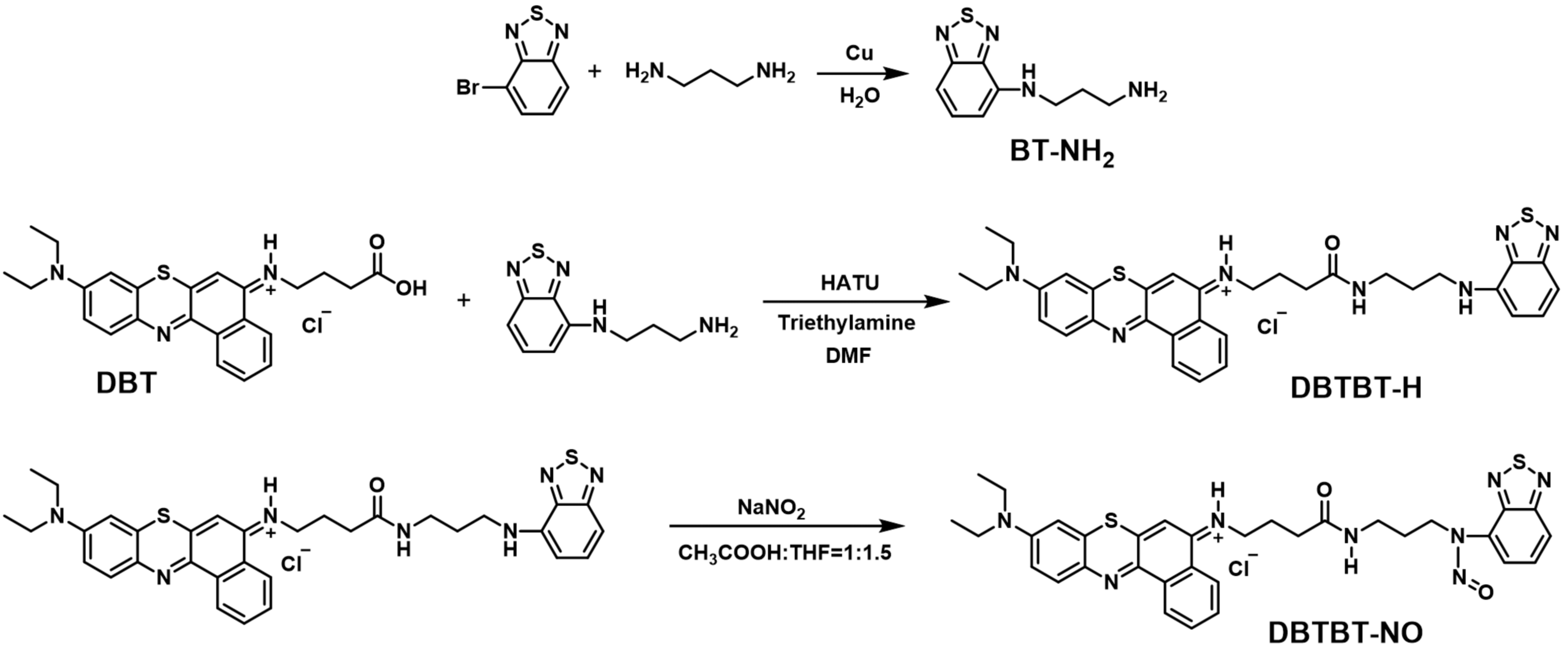


**Figure S1**. Synthetic route of DBTBT-NO.


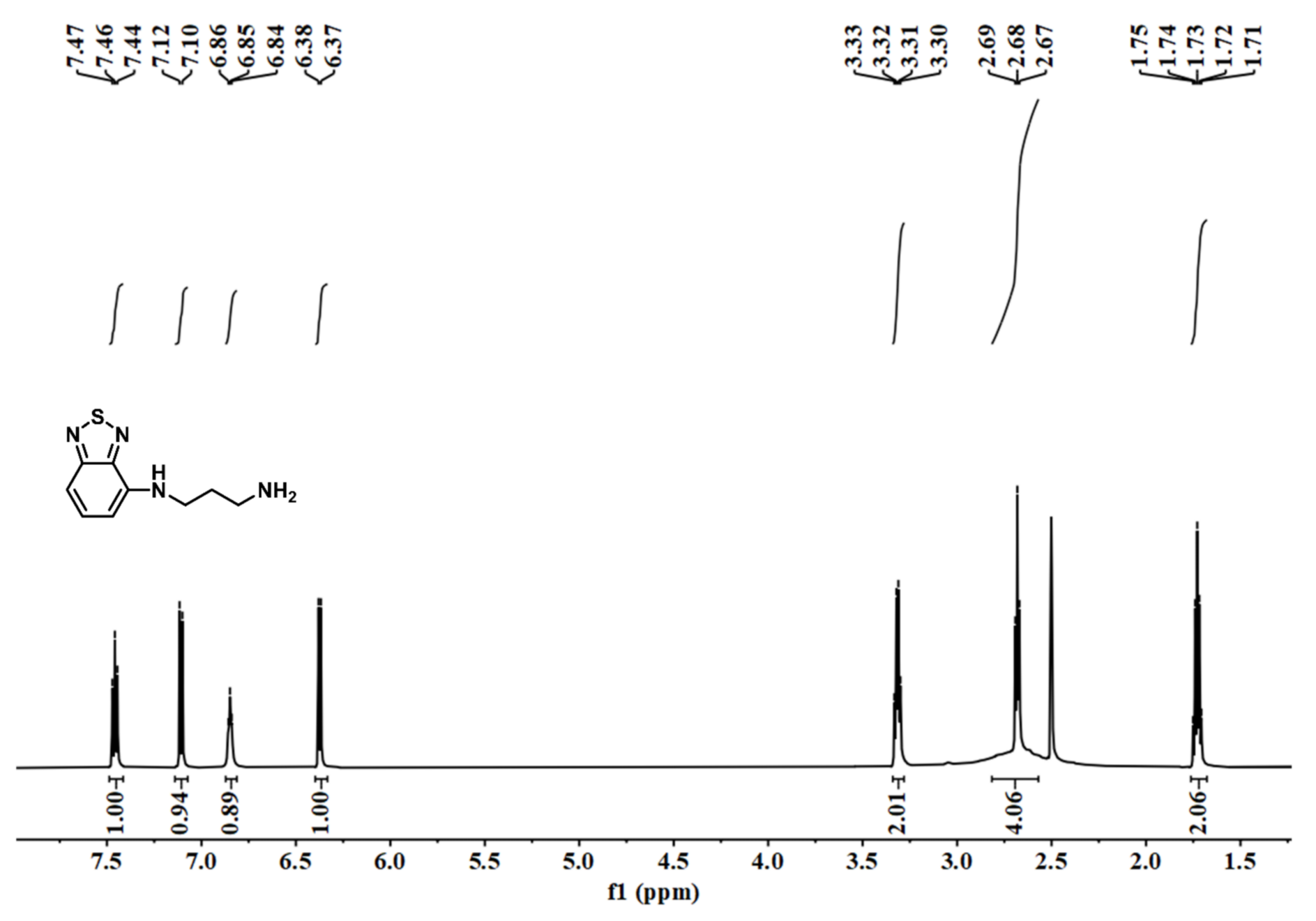


**Figure S2.** ^1^ H NMR spectrum of BT-NH_2_.


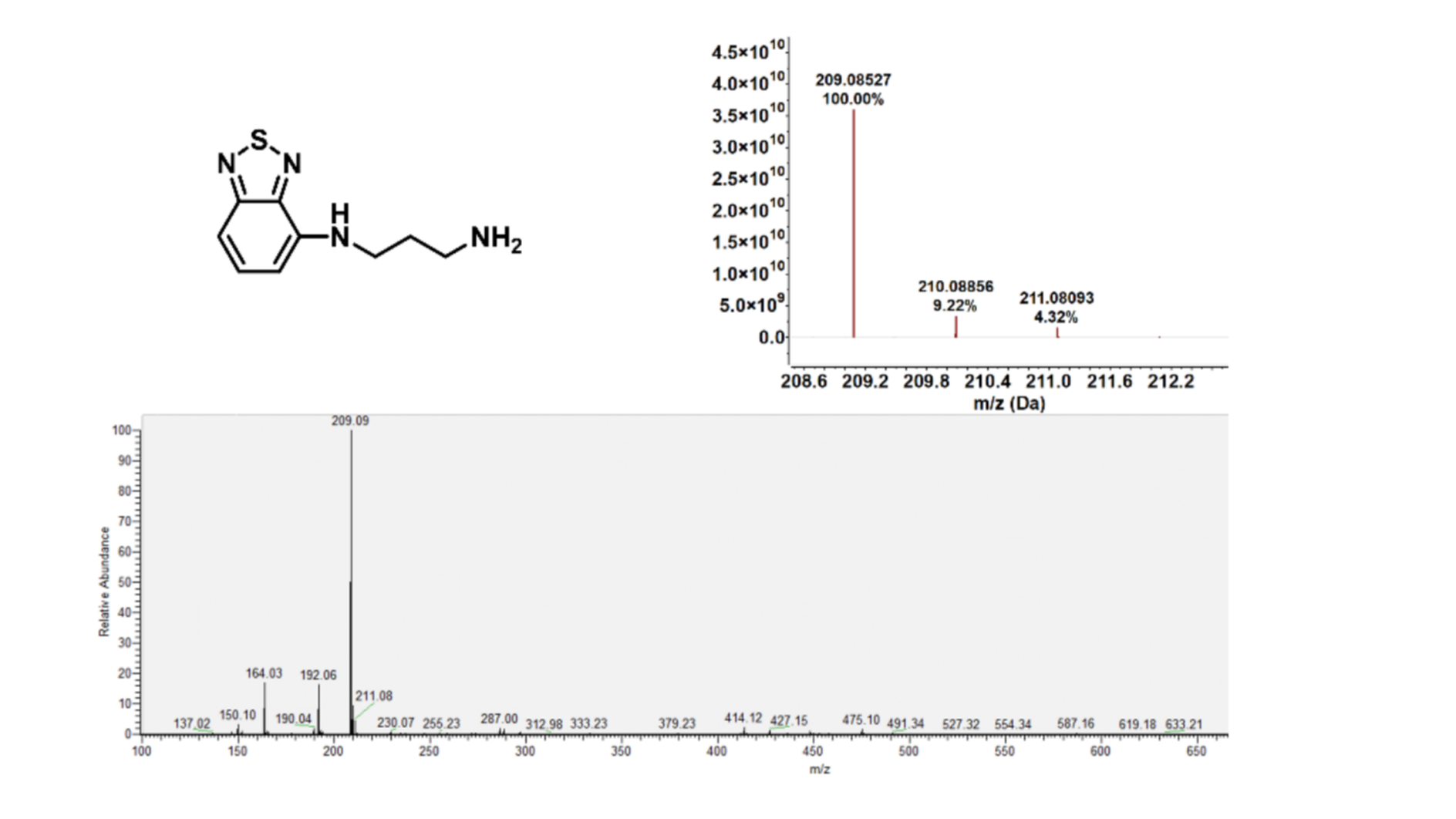


**Figure S3.** HR-MS of BT-NH_2_.


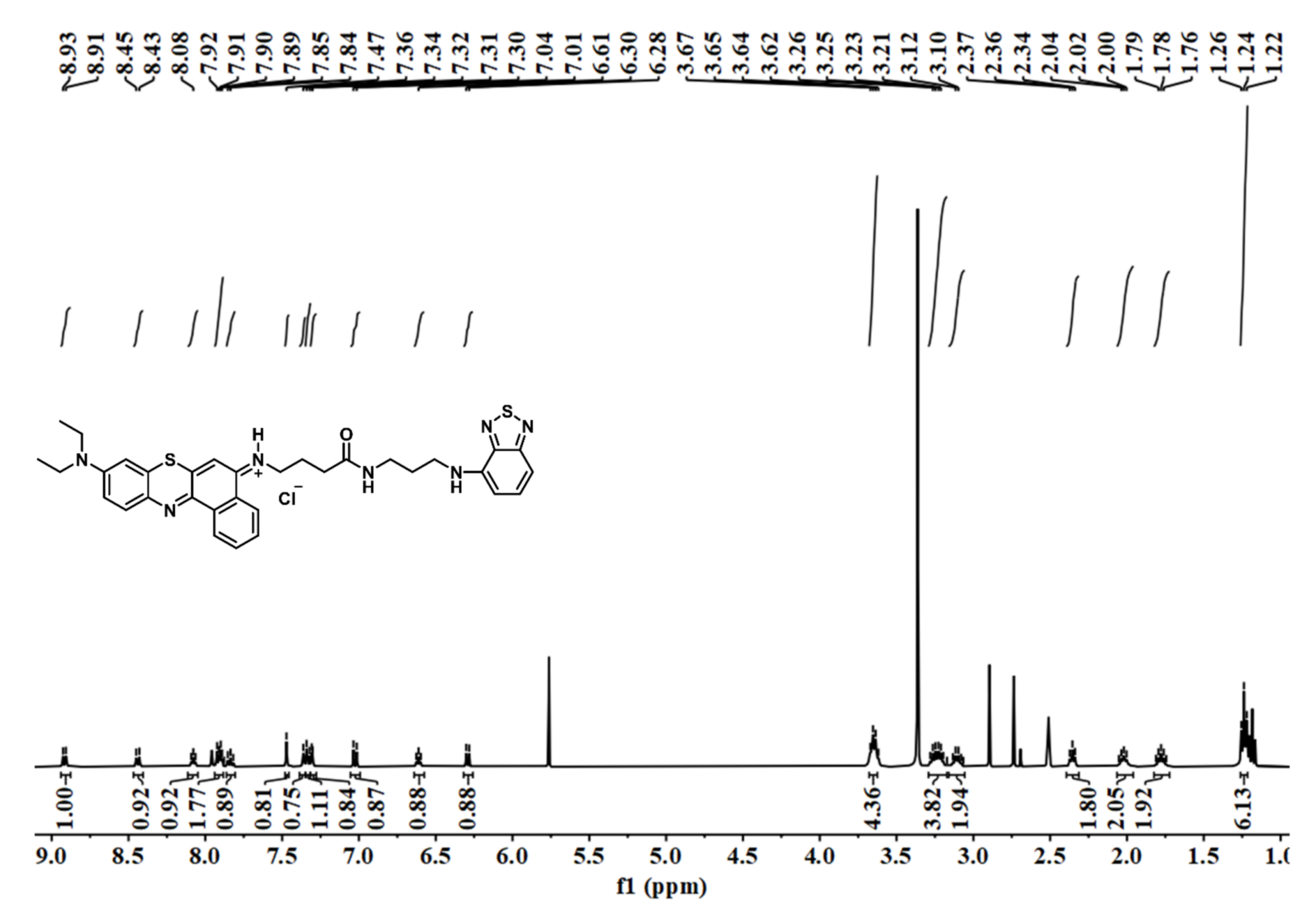


**Figure S4.** ^1^ H NMR spectrum of DBTBT-H.


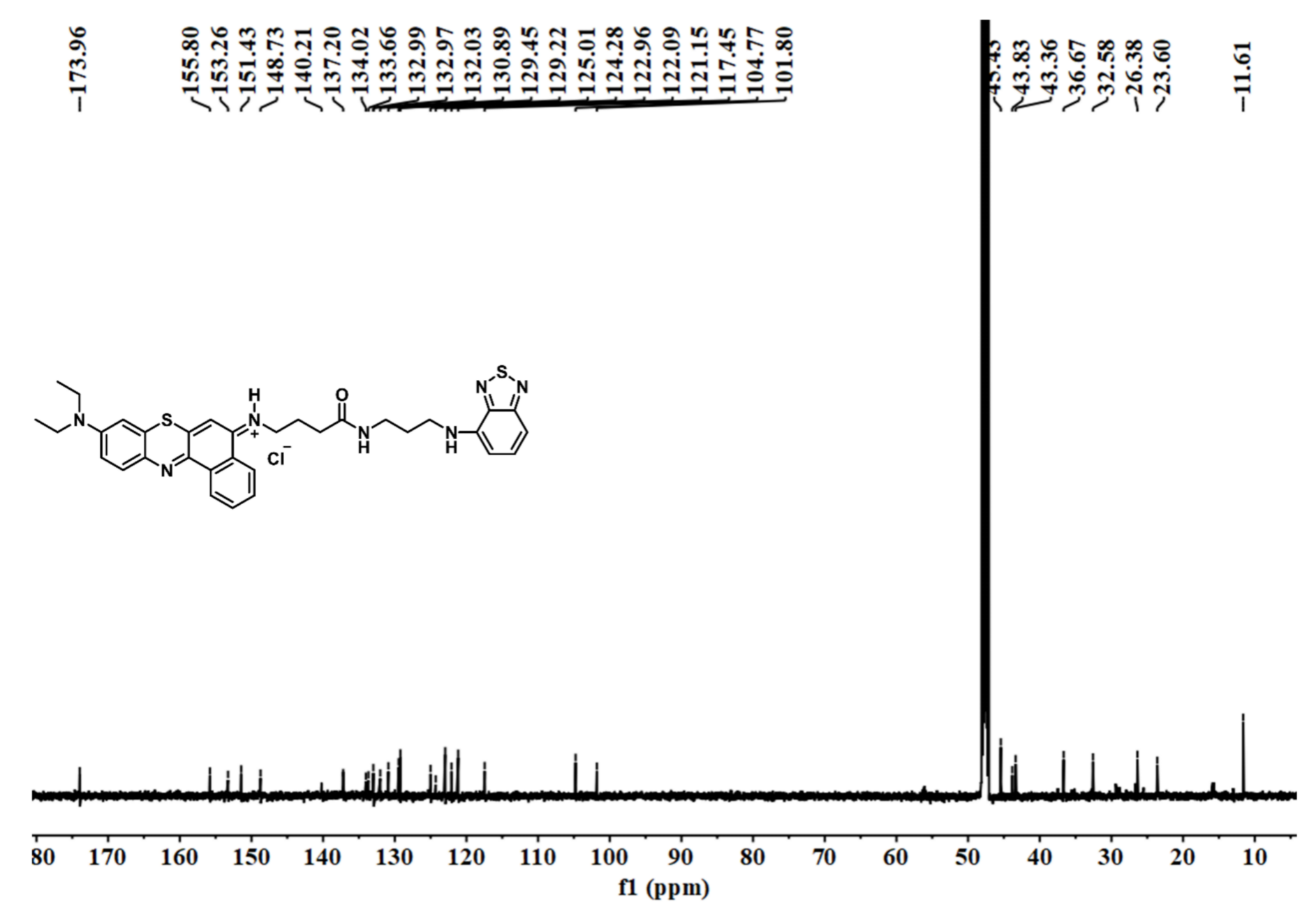


**Figure S5.** ^13^ C NMR spectrum of DBTBT-H.


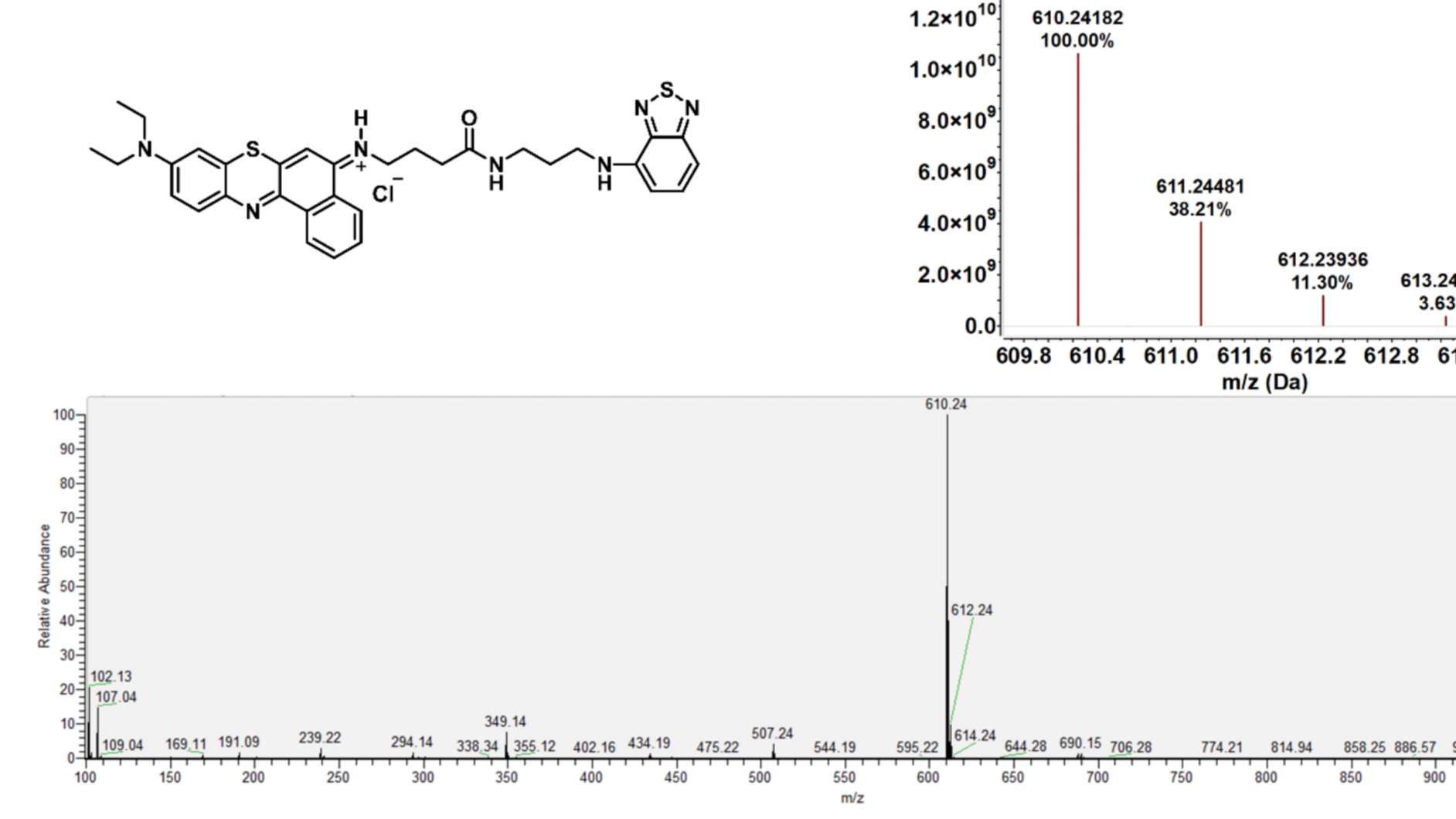


**Figure S6.** HR-MS of DBTBT-H.


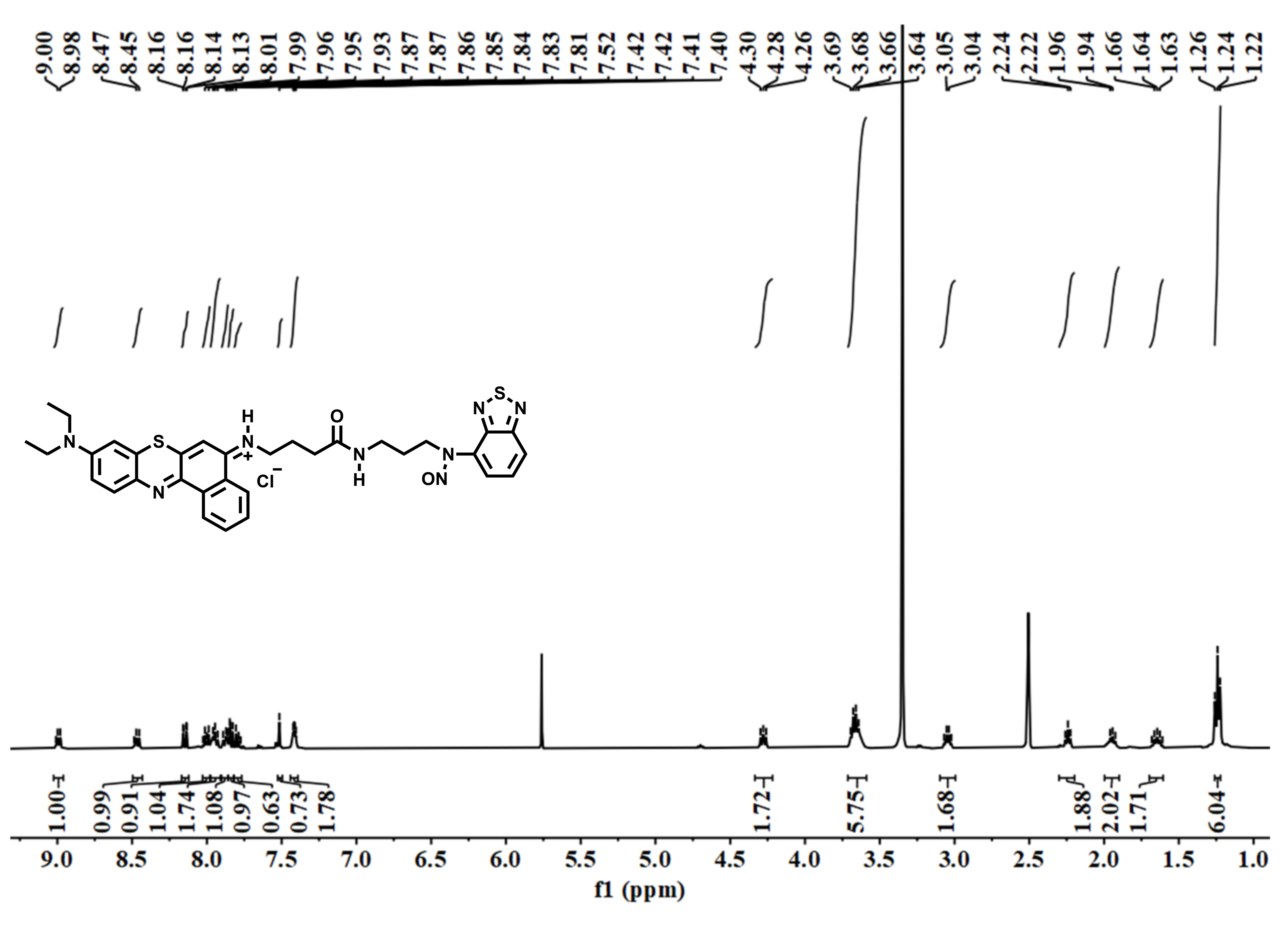


**Figure S7.** ^1^ H NMR spectrum of DBTBT-NO.


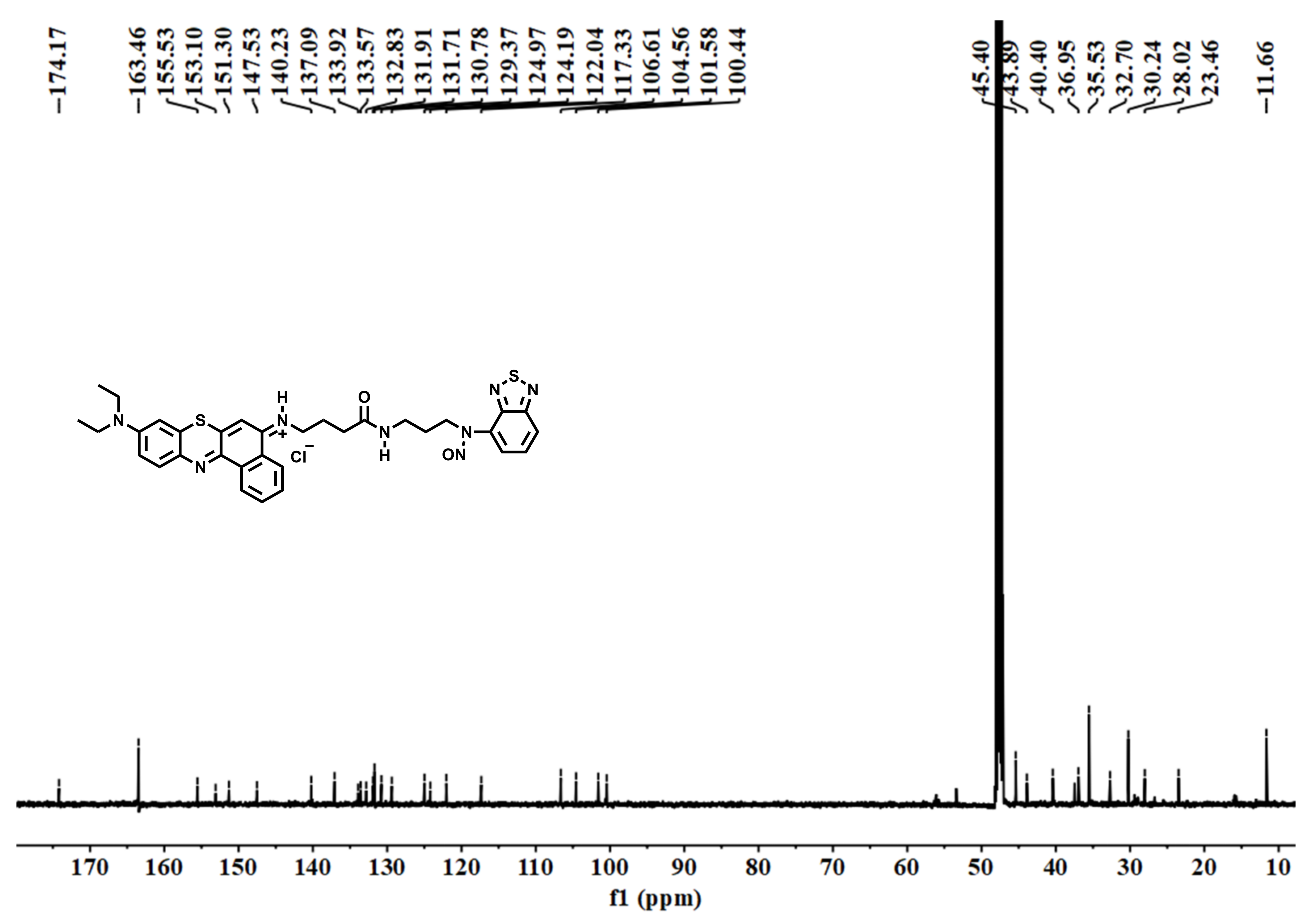


**Figure S8.** ^13^ C NMR spectrum of DBTBT- NO.


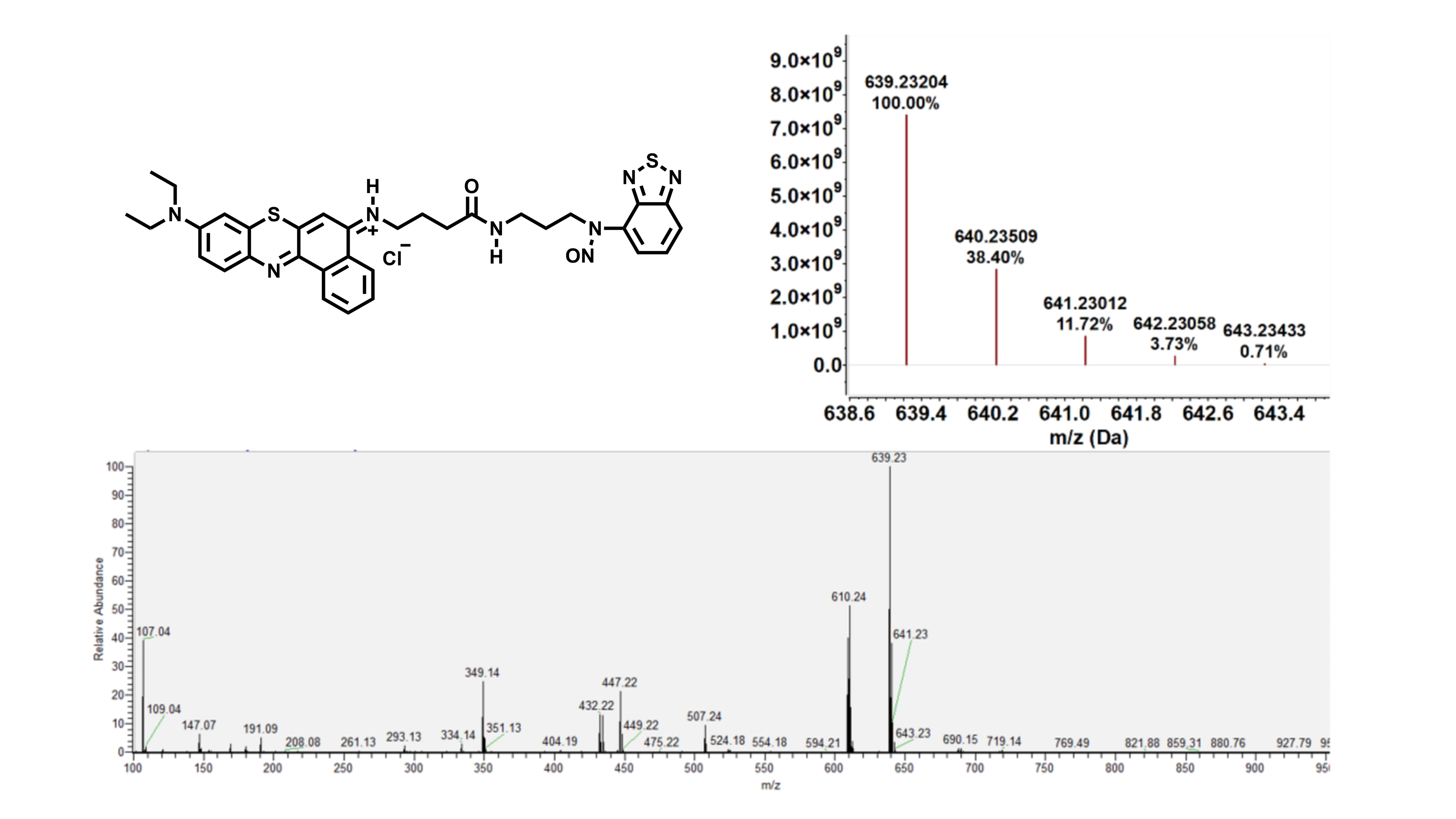


**Figure S9.** HR-MS of DBTBT- NO.


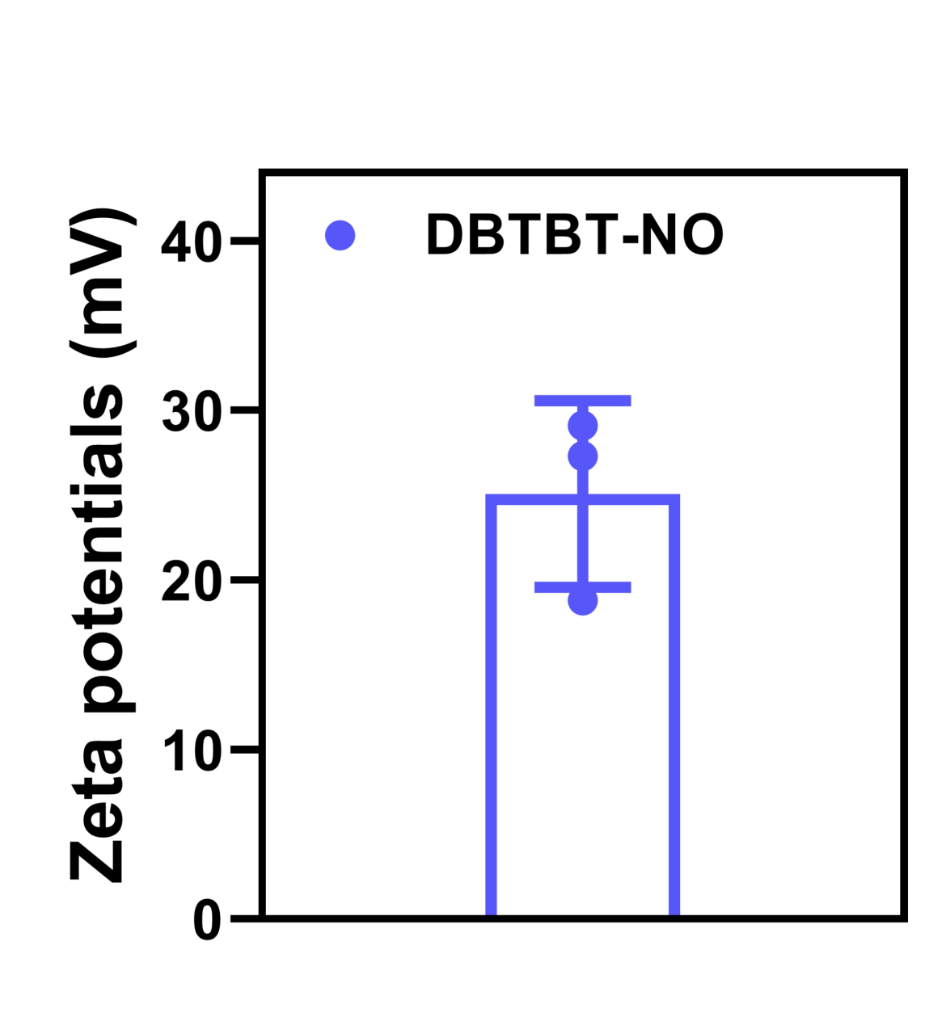


**Figure S10.** Zeta potentials of DBTBT- NO in aqueous solution.


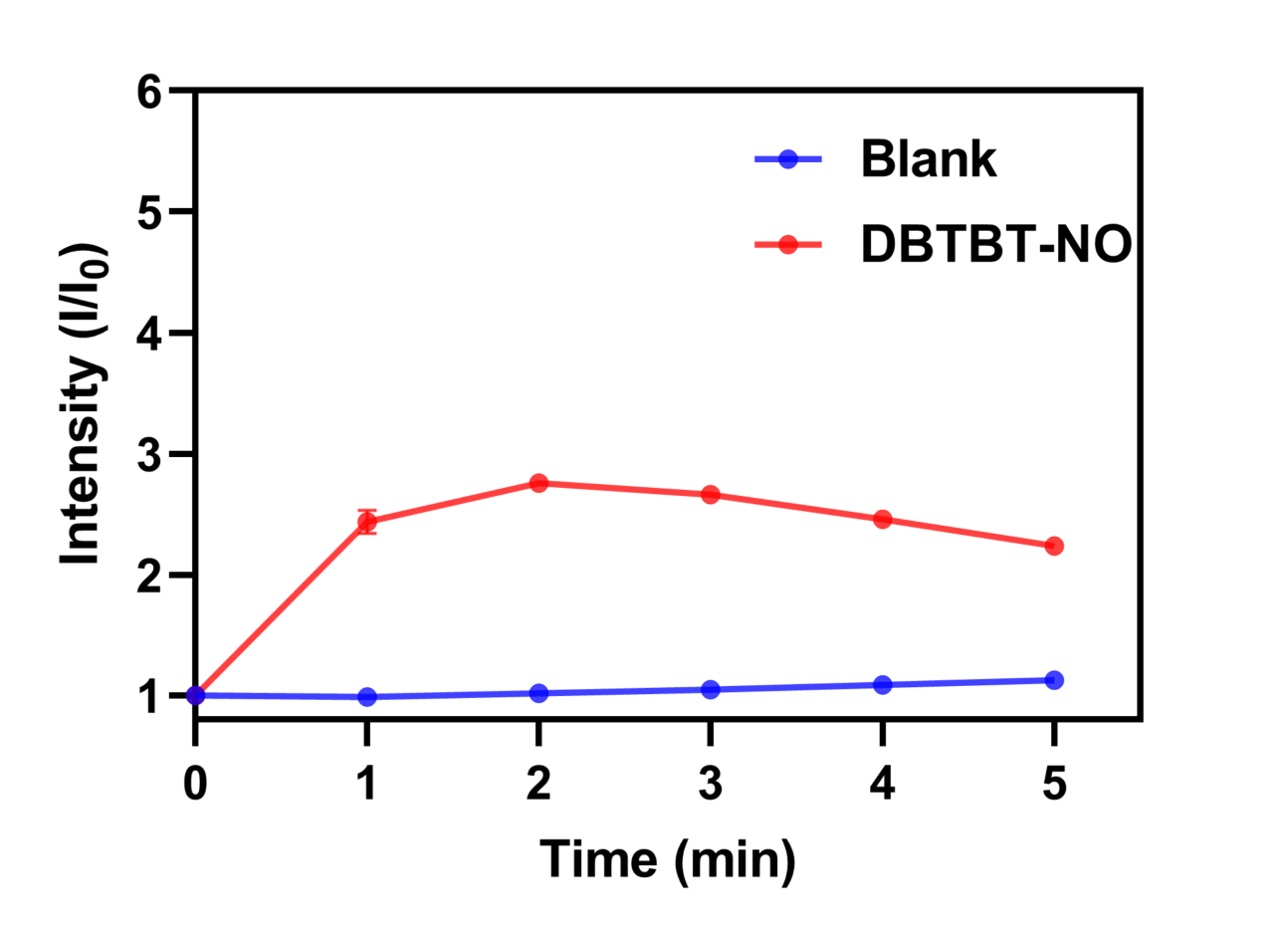


**Figure S11.** The superoxide anion generation of DBTBT- NO in aqueous solution after light irradiation.


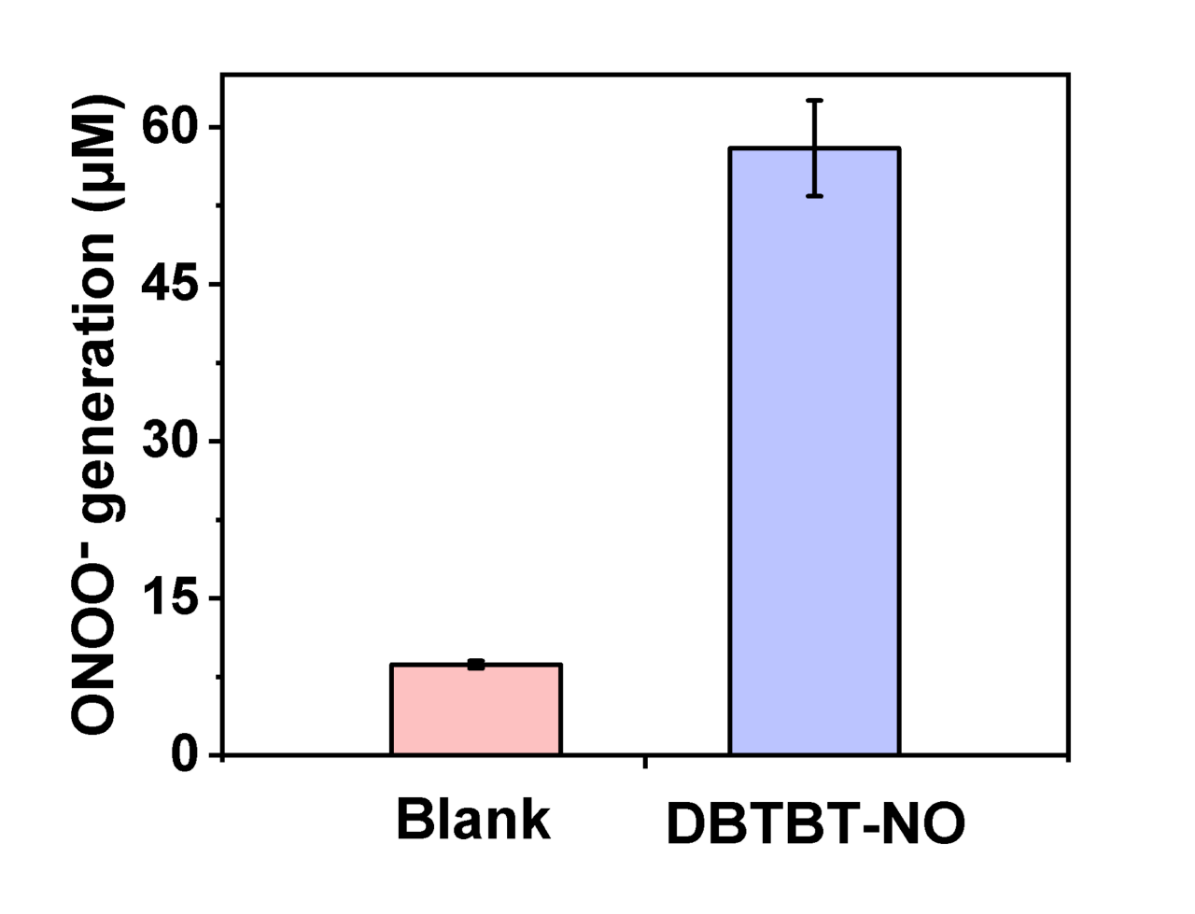


**Figure S12.** The quantitative measurement of peroxynitrite generated from DBTBT- NO in aqueous solution after light irradiation.


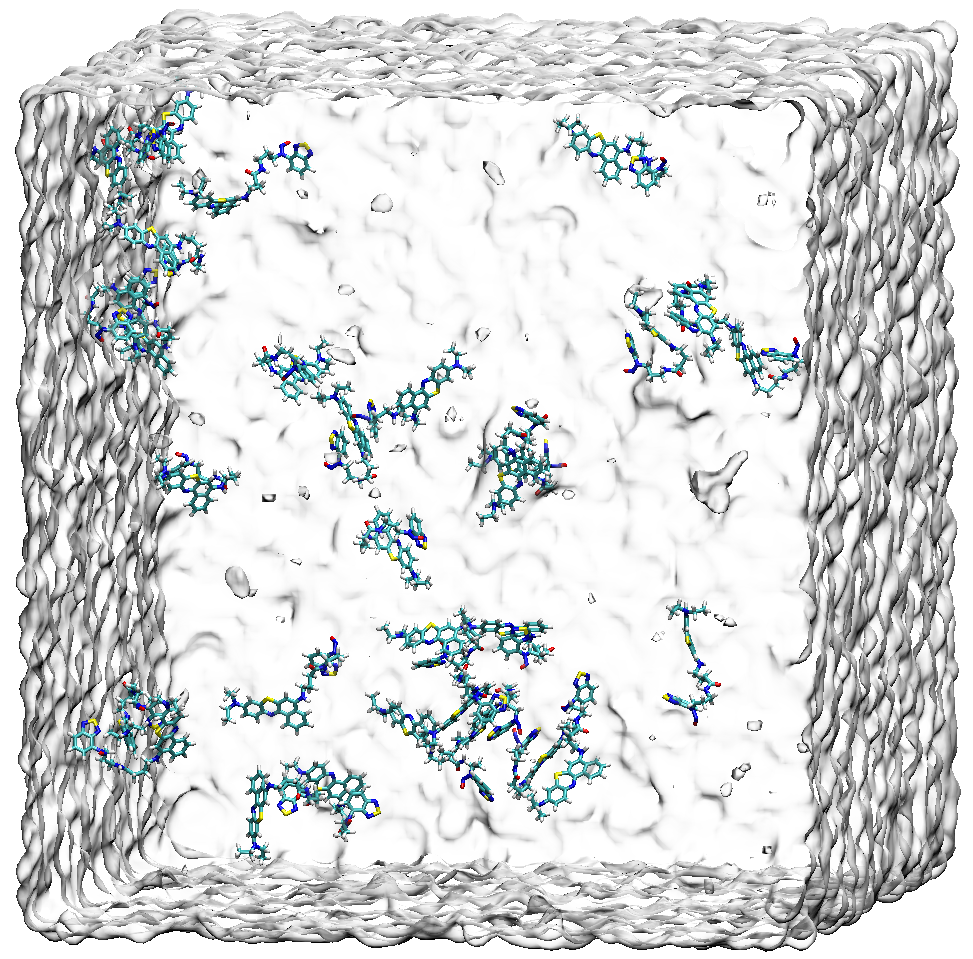


**Figure S13.** The random distribution of DBTBT-NO at the initial state in MD simulations.


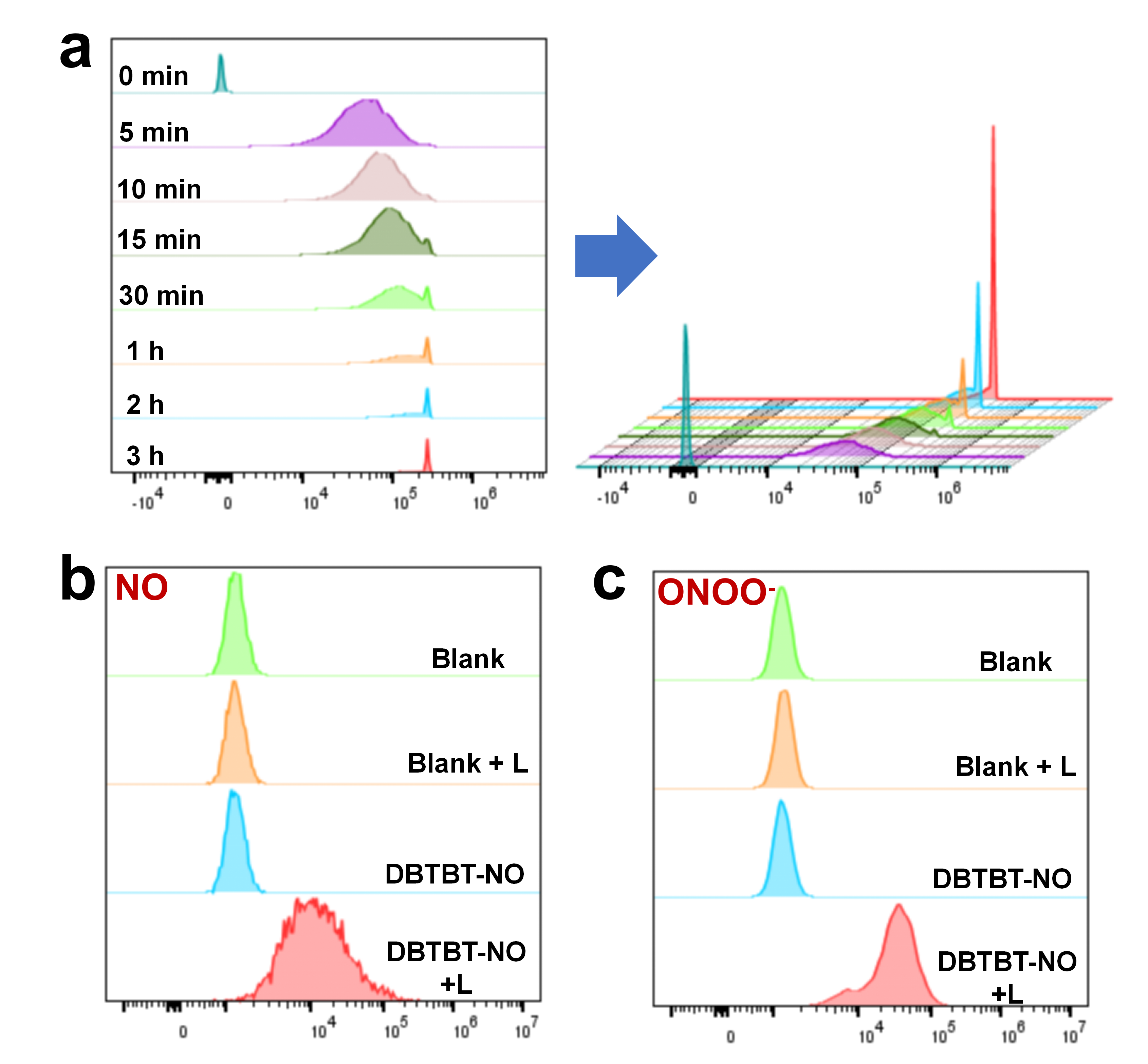


**Figure S14.** The flow cytometry analysis about a) cellular uptake, b) NO production, and c) ONOO^-^ generation abilities of DBTBT-NO in 4T1 cells.


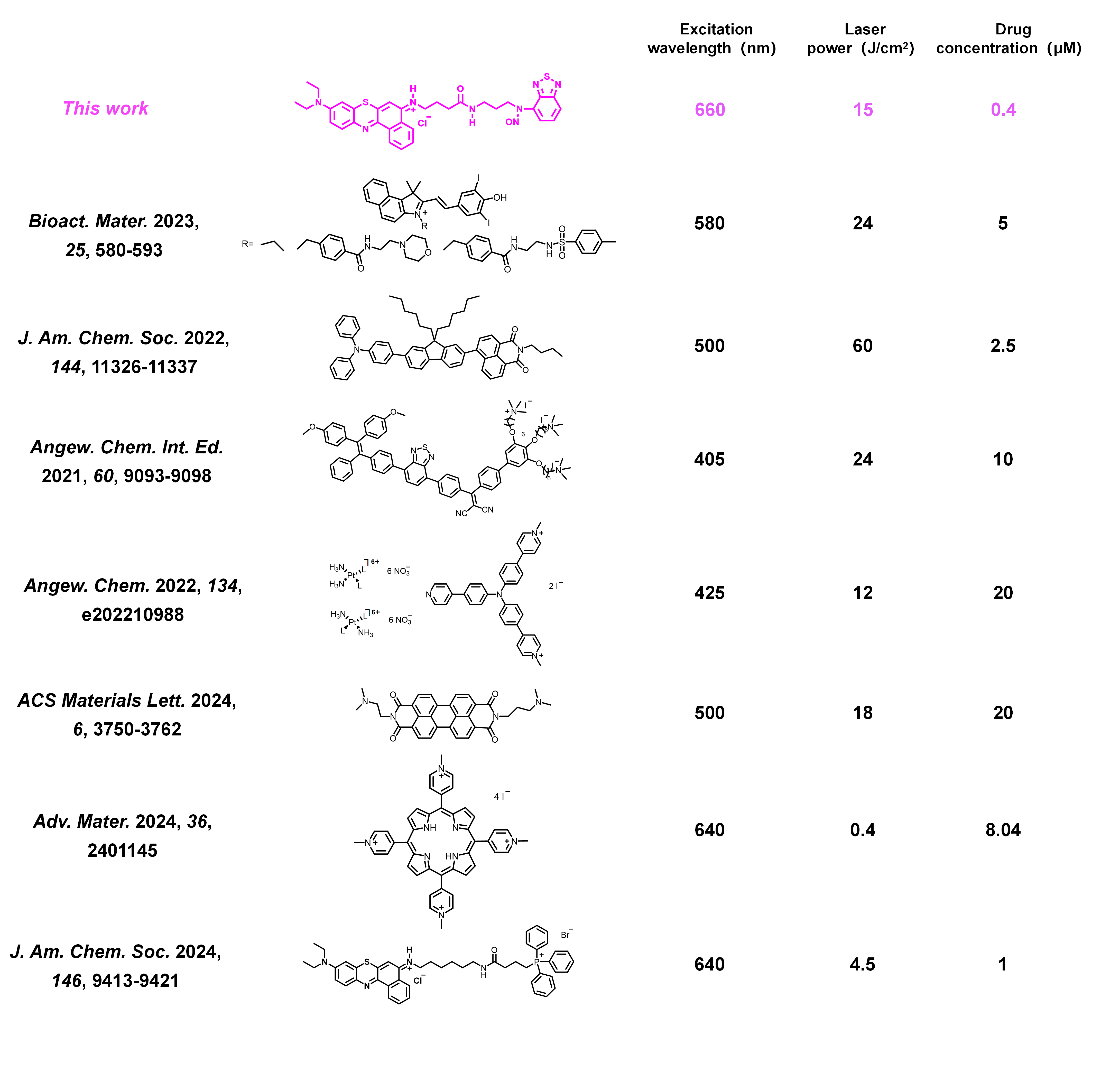


**Figure S15.** Comparison of pyroptosis efficiency with reported literatures.


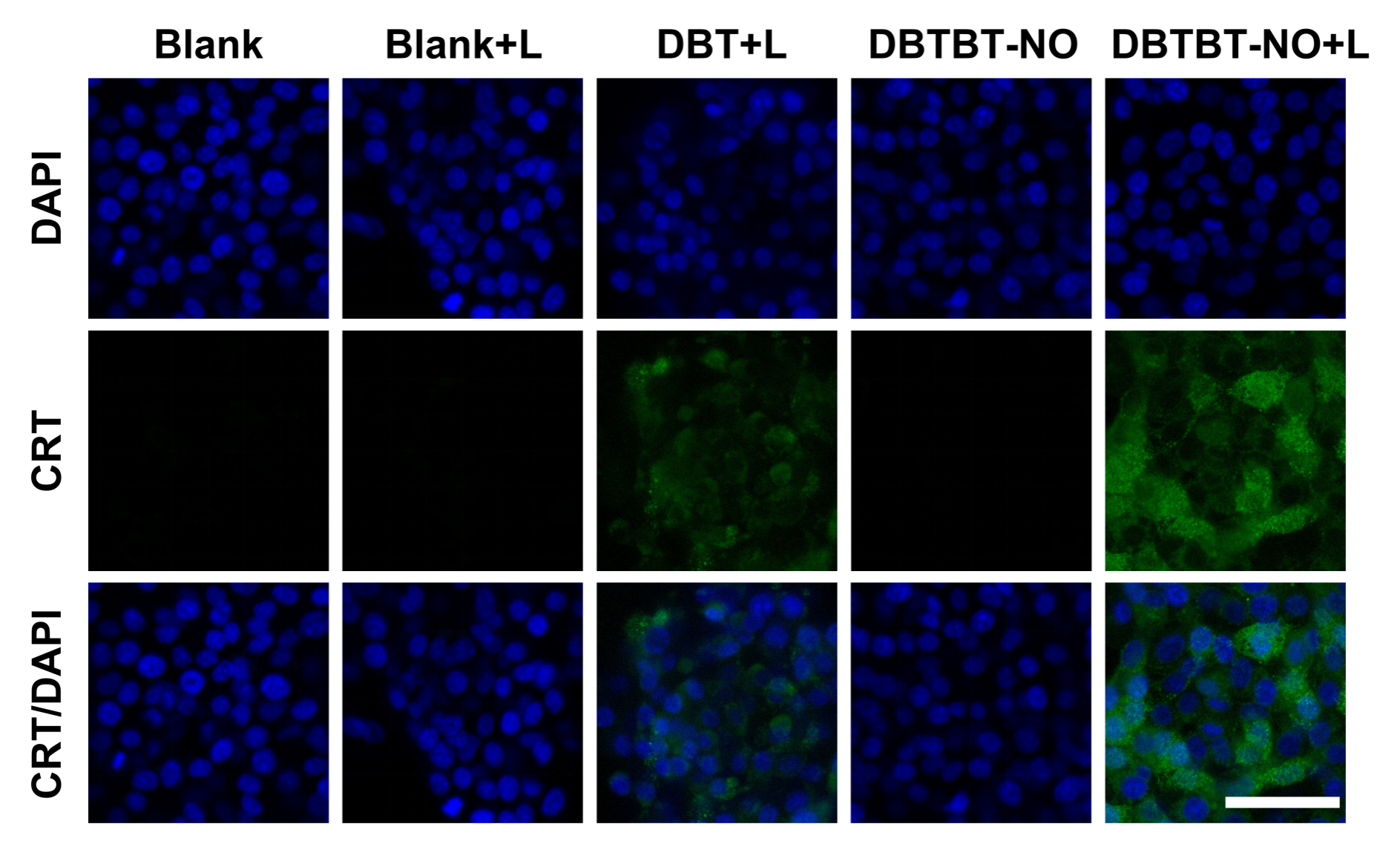


**Figure S16.** CLSM images of CRT on 4T1 cells stained with the anti-CALR antibody after different treatments. The cell nucleus was stained with DAPI.


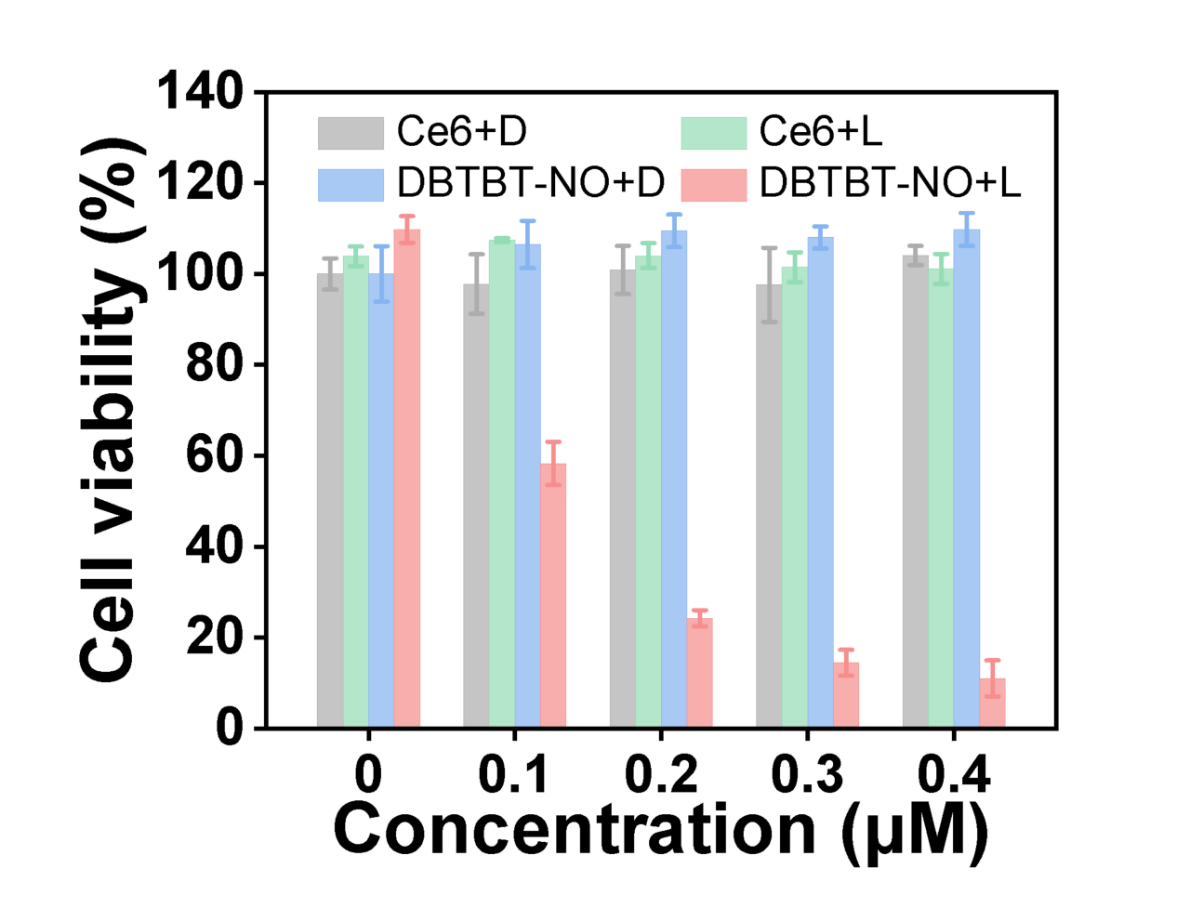


**Figure S17.** Cell viability of 4T1 cells respectively treated by Ce6 and DBTBT-NO in the presence or the absence of light.


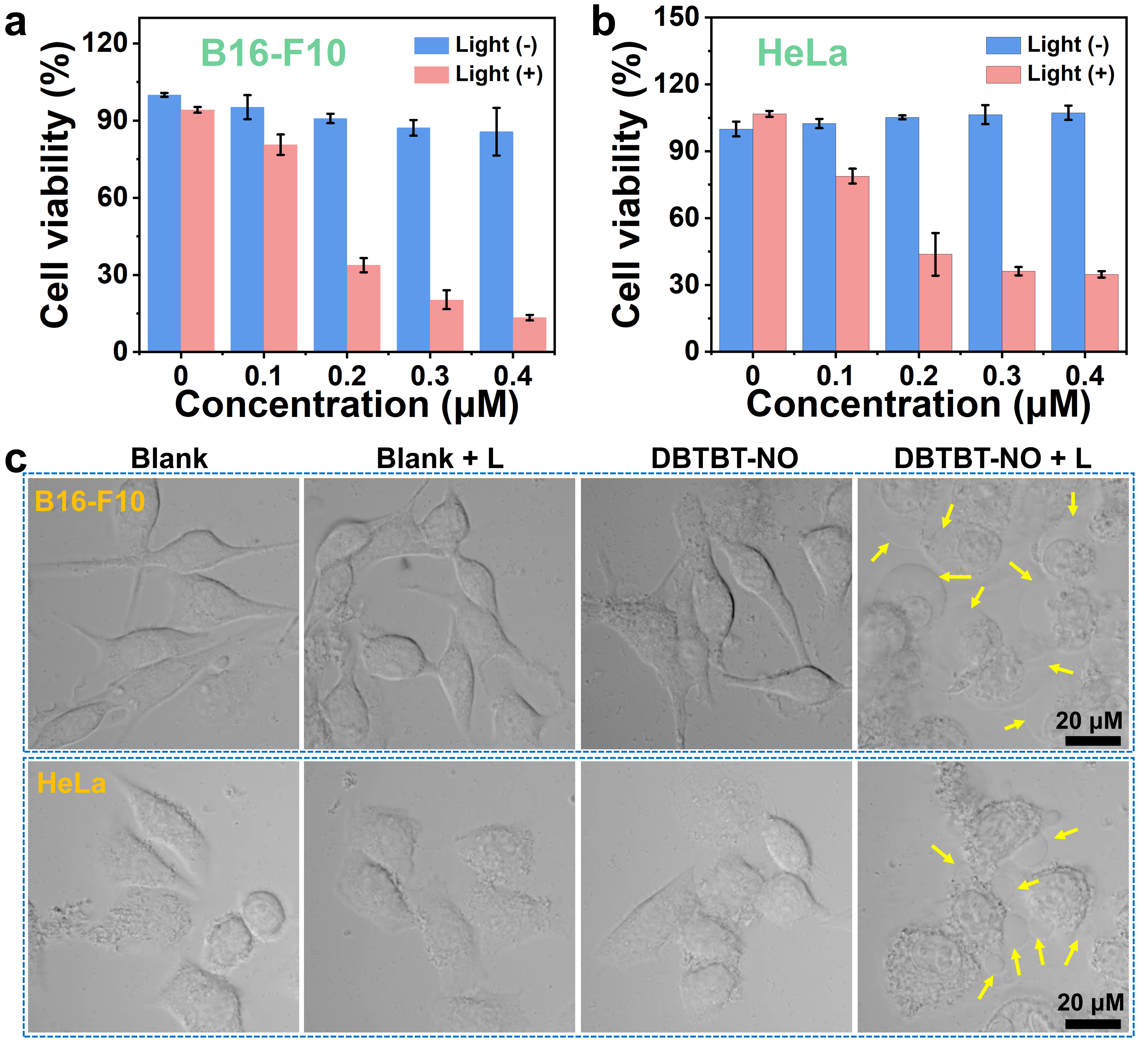


**Figure S18.** Cell ability of a) B16-F10 cells and b) HeLa cells incubated with different concentrations of DBTBT-NO in dark or light (660 nm, 0.25 W/cm^2^, 3 min). c) Cell morphological change (bright field) of B16-F10 and HeLa cells after different treatments (660 nm, 0.25 W/cm^2^, 1 min). The yellow arrows indicated the cell bubbling.


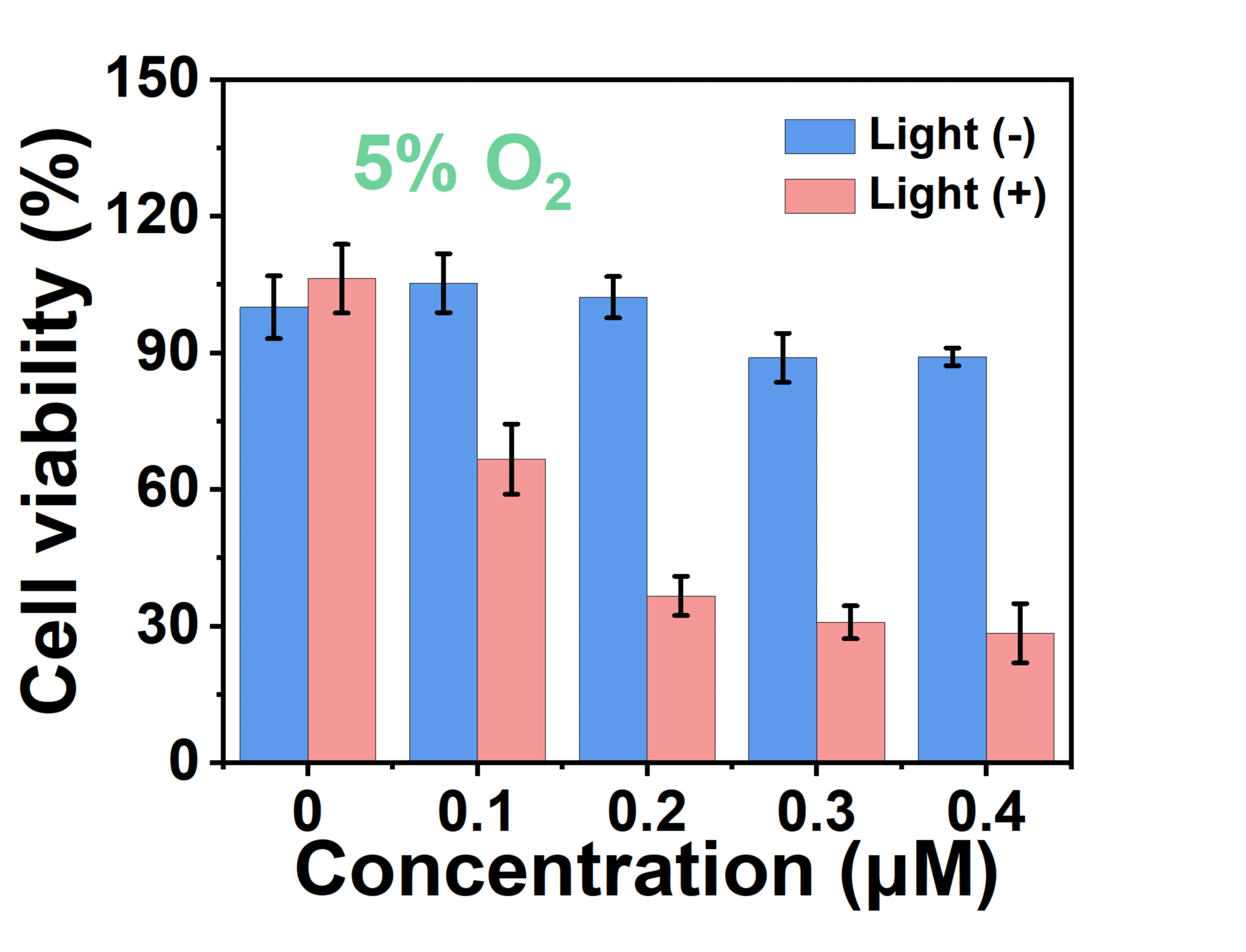


**Figure S19.** Cell ability of 4T1 cells incubated with different concentrations of DBTBT-NO in dark or light (660 nm, 0.25 W/cm^2^, 3 min) under hypoxic conditions.


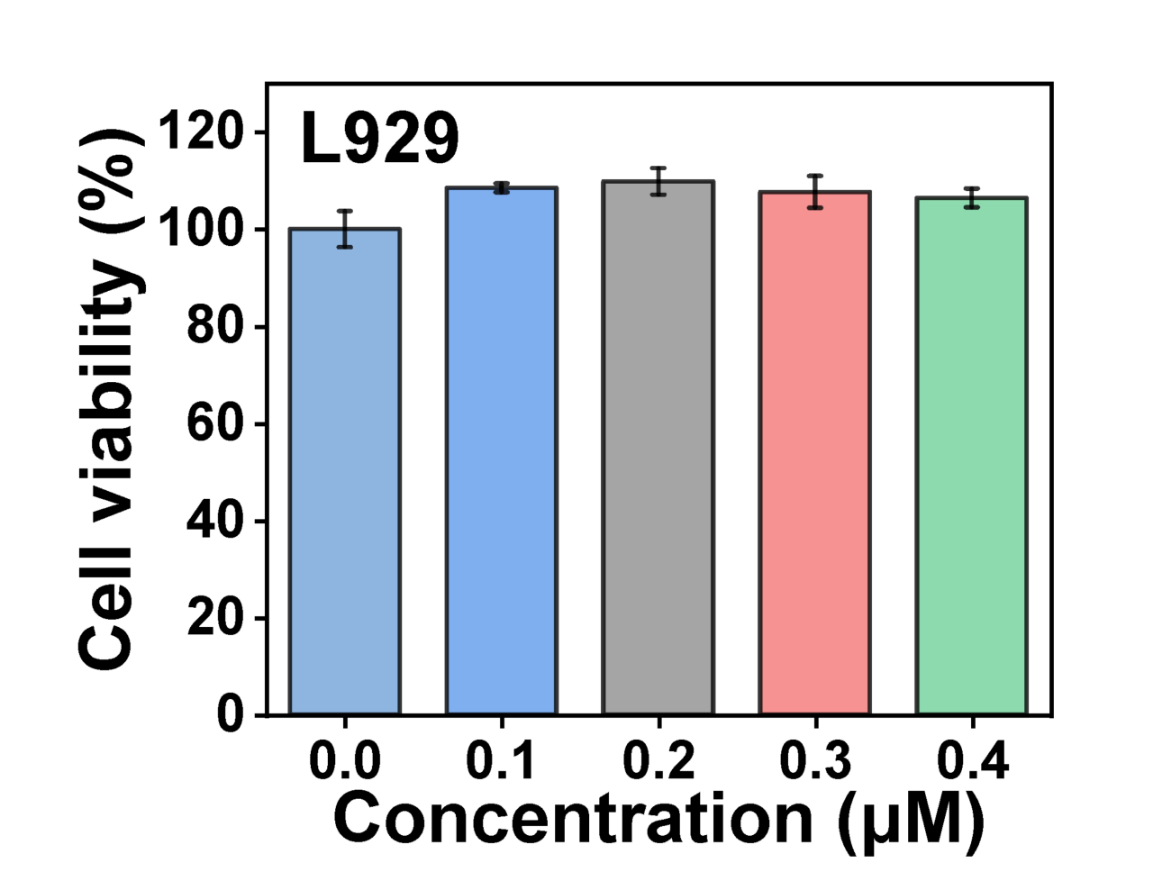


**Figure S20.** Cell viability of normal cells L929 treated by DBTBT-NO.


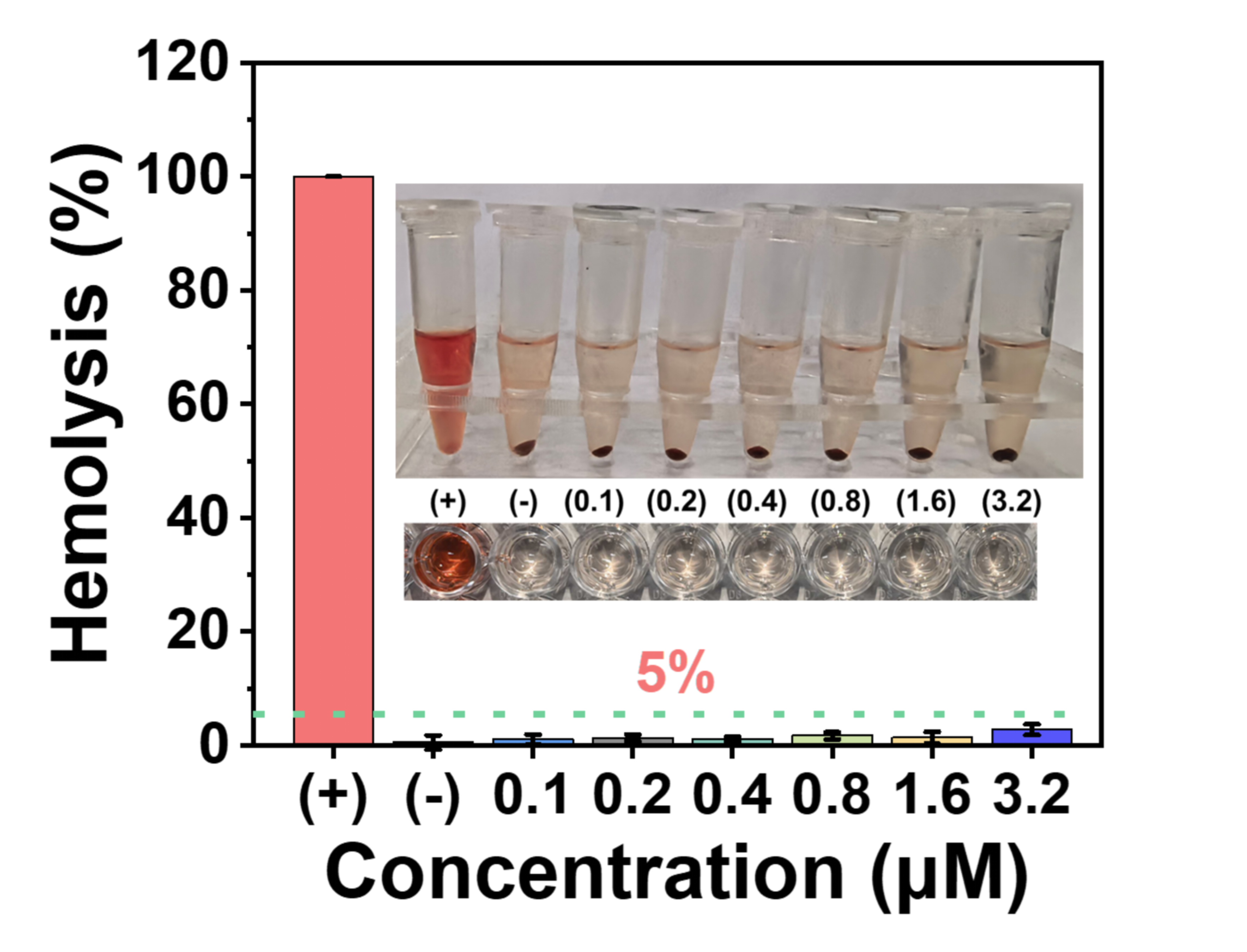


**Figure S21.** Hemolysis ratio and photographs of hemolytic activity assay of DBTBT-NO.


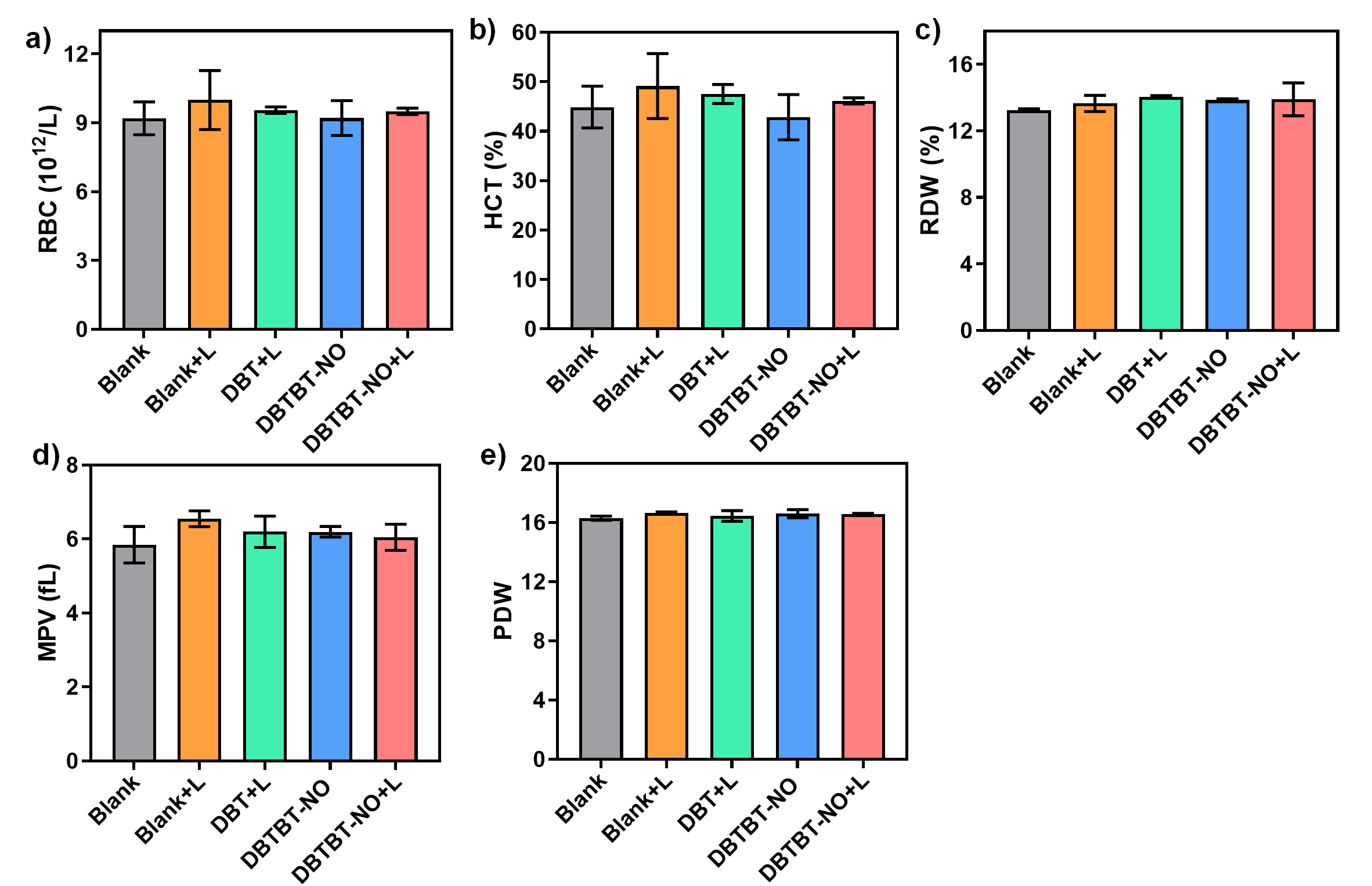


**Figure S22.** Blood routine examination of tumor-bearing mice after different treatments. Data are presented as mean±SD (n=3).


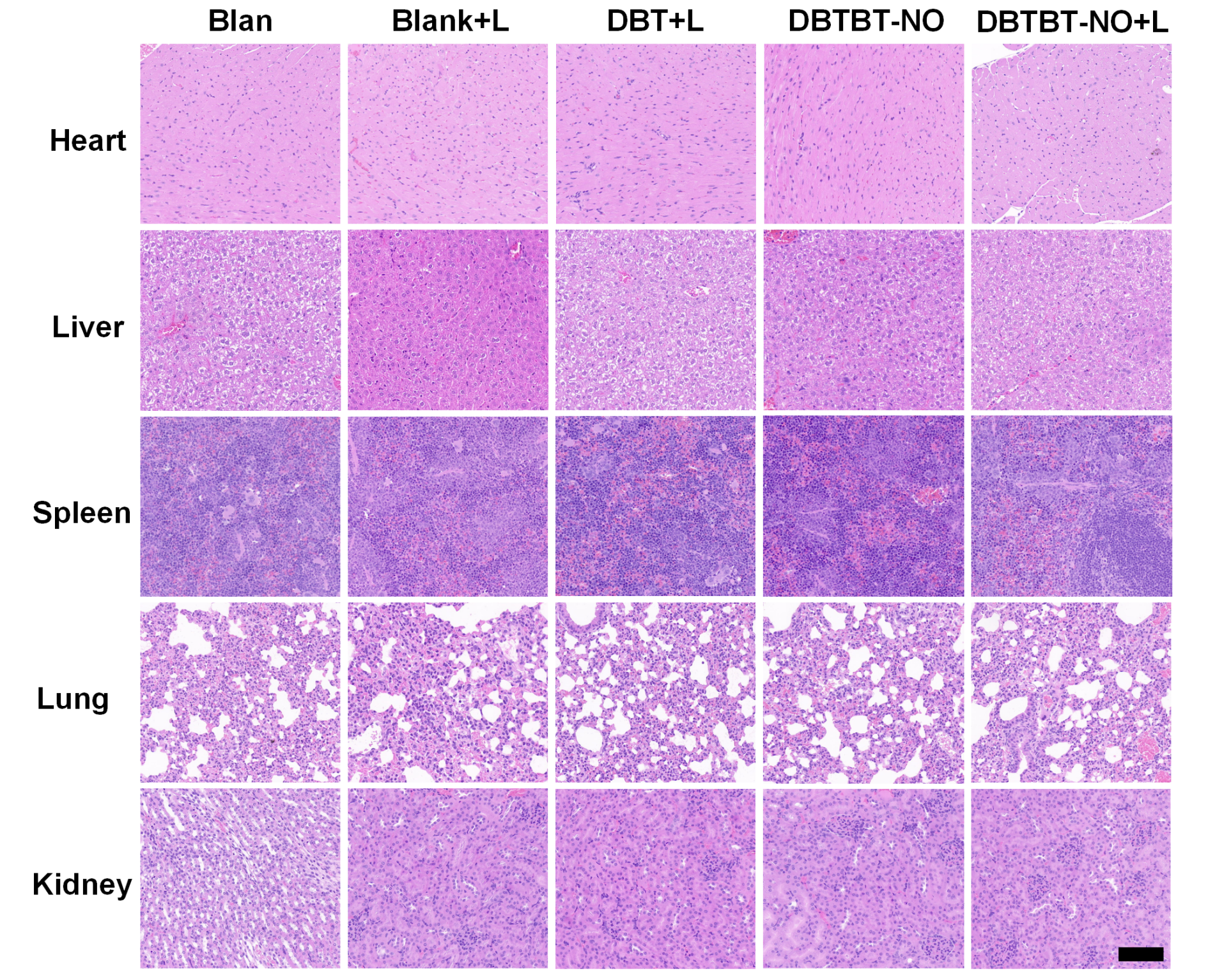


**Figure S23.** H&E staining of major organs of mice after different treatments.


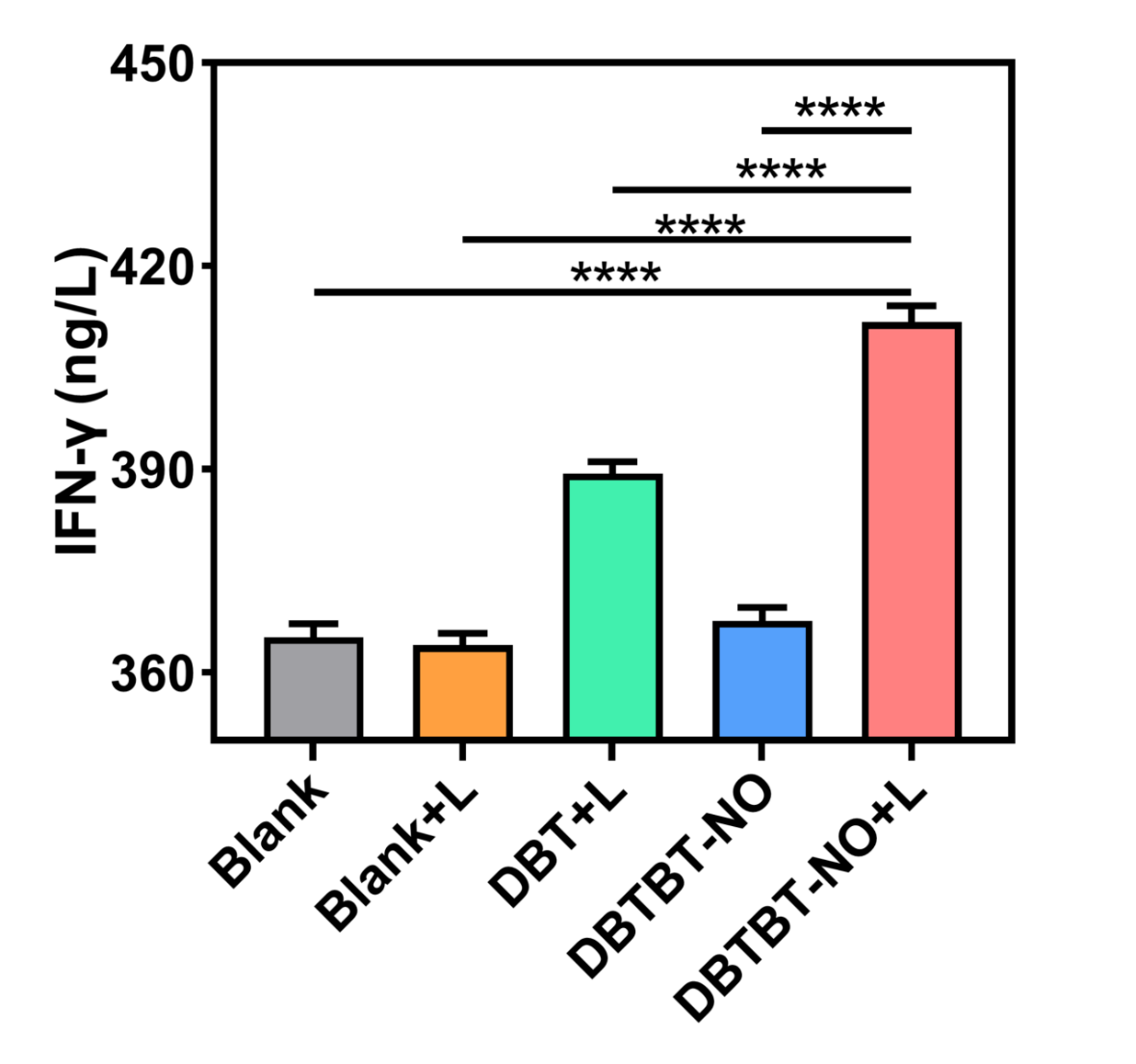


**Figure S24.** Levels of IFN-γ in serum of mice after various treatments.


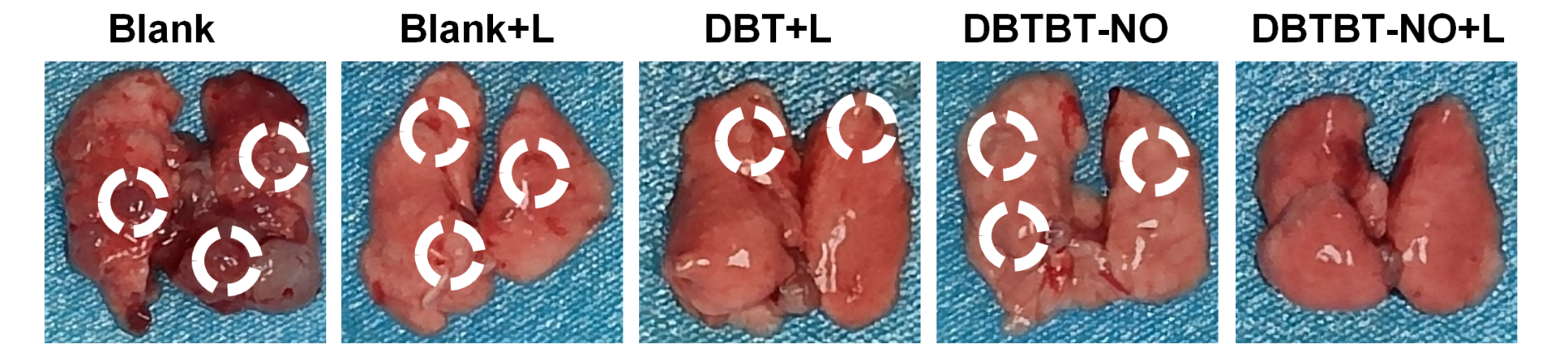


**Figure S25.** Representative photographs of lungs of mice showing the distribution of tumor nodules after different treatments.
